# Diverse Splicing Patterns of Exonized Alu Elements in Human Tissues

**DOI:** 10.1371/journal.pgen.1000225

**Published:** 2008-10-17

**Authors:** Lan Lin, Shihao Shen, Anne Tye, James J. Cai, Peng Jiang, Beverly L. Davidson, Yi Xing

**Affiliations:** 1 Department of Internal Medicine, University of Iowa, Iowa City, Iowa, United States of America; 2 Department of Biostatistics, University of Iowa, Iowa City, Iowa, United States of America; 3 Department of Biology, Stanford University, Stanford, California, United States of America; 4 Department of Molecular Physiology and Biophysics, University of Iowa, Iowa City, Iowa, United States of America; 5 Department of Neurology, University of Iowa, Iowa City, Iowa, United States of America; 6 Department of Biomedical Engineering, University of Iowa, Iowa City, Iowa, United States of America; RIKEN Genomic Sciences Center, Japan

## Abstract

Exonization of Alu elements is a major mechanism for birth of new exons in primate genomes. Prior analyses of expressed sequence tags show that almost all Alu-derived exons are alternatively spliced, and the vast majority of these exons have low transcript inclusion levels. In this work, we provide genomic and experimental evidence for diverse splicing patterns of exonized Alu elements in human tissues. Using Exon array data of 330 Alu-derived exons in 11 human tissues and detailed RT-PCR analyses of 38 exons, we show that some Alu-derived exons are constitutively spliced in a broad range of human tissues, and some display strong tissue-specific switch in their transcript inclusion levels. Most of such exons are derived from ancient Alu elements in the genome. In SEPN1, mutations of which are linked to a form of congenital muscular dystrophy, the muscle-specific inclusion of an Alu-derived exon may be important for regulating SEPN1 activity in muscle. Realtime qPCR analysis of this SEPN1 exon in macaque and chimpanzee tissues indicates human-specific increase in its transcript inclusion level and muscle specificity after the divergence of humans and chimpanzees. Our results imply that some Alu exonization events may have acquired adaptive benefits during the evolution of primate transcriptomes.

## Author Summary

New exons have been created and added to existing functional genes during eukaryotic genome evolution. Alu elements, a class of primate-specific retrotransposons, are a major source of new exons in primates. However, recent analyses of expressed sequence tags suggest that the vast majority of Alu-derived exons are low-abundance splice forms and represent non-functional evolutionary intermediates. In order to elucidate the evolutionary impact of Alu-derived exons, we investigated the splicing of 330 Alu-derived exons in 11 human tissues using data from high-density exon arrays with multiple oligonucleotide probes for every exon in the human genome. Our exon array analysis and further RT-PCR experiments reveal surprisingly diverse splicing patterns of these exons. Some Alu-derived exons are constitutively spliced, and some are strongly tissue-specific. In SEPN1, a gene implicated in a form of congenital muscular dystrophy, our data suggest that the muscle-specific inclusion of an Alu-derived exon results from a human-specific splicing change after the divergence of humans and chimpanzees. Our study provides novel insight into the evolutionary significance of Alu exonization events. A subset of Alu-derived exons, especially those derived from more ancient Alu elements in the genome, may have contributed to functional novelties during primate evolution.

## Introduction

Alu is a class of primate-specific transposable elements that belongs to the short interspersed nuclear elements (SINE) family [1]. The rapid expansion of Alu during primate evolution has produced over one million copies of Alu elements in the human genome [2]. Until recently, Alu elements were considered as “junk DNA”, with no important functional or regulatory roles [1]. However, recent studies suggest a substantial influence by Alu elements on evolution of the human genome and regulation of gene expression [3].

Alu is a major source of new exons in primate genomes [4]–[6]. Alu elements have several sites resembling consensus splice sites in both sense and antisense orientations [7]. Therefore, the insertion of Alu elements into intronic regions may introduce new exons into existing, functioning genes. The evolutionary history of several such “exonization” events has been characterized in detail [8],[9]. For example, in p75TNFR, the insertion of an Alu element and a series of subsequent nucleotide substitutions created a new alternative first exon [8]. Sorek and colleagues investigated the splicing pattern of 61 Alu-containing exons using human mRNA and EST sequences [4]. All Alu-containing exons were alternatively spliced. The vast majority of these exons were included in the minor transcript isoforms, based on ESTs pooled from all tissues [4]. This is consistent with the hypothesis that the creation of a new minor-form alternative exon reduces the initial deleterious effects of exonization events [10]. However, due to the high noise in EST sequencing [11] and the low EST coverage for these Alu-derived exons [4], it was difficult to assess the splicing patterns of individual exons tissue by tissue. Regardless, there have been anecdotal reports for Alu-containing exons to have splicing patterns other than minor-form alternative splicing. Based on the tissue origins of human EST sequences, Mersch et al. predicted a few Alu-containing exons to be tissue-specific [12]. In another study, an Alu-containing exon of FAM55C was shown to be constitutively spliced in a neuroblastoma cell line [13]. These data suggest that the splicing profiles of exonized Alu elements may be more diverse than previously expected. In this study, we combined a genome-scale Exon array analysis with RT-PCR experiments to investigate the splicing profiles of exonized Alu elements in human tissues.

## Results

### Splicing Signal and Evolutionary Rate of Alu-Derived Exons

We collected a list of 330 Alu-derived exons, using annotations from the UCSC Genome Browser database [14] and Affymetrix human Exon 1.0 arrays (see details in Materials and Methods). We first analyzed the splicing signals of these exons as well as their evolutionary rates during primate evolution. For the purpose of comparison, we also analyzed 13103 constitutively spliced exons and 5389 exon-skipping cassette exons in the human genome, which were collected after applying a set of stringent filtering criteria to exons in the Alternative Splicing Annotation Project 2 (ASAP2) database (see Materials and Methods).

Our analysis showed that Alu-derived exons had significantly weaker splicing signals compared to constitutively spliced exons and typical cassette exons. For each exon, we scored its 5′ and 3′ splice site using models of consensus splice sites in MAXENT [15]. The median 5′ splice site score of Alu-derived exons was 7.35, compared to 8.27 for cassette exons and 8.88 for constitutive exons, a statistically significant difference (P = 3.0e-6 for Alu-derived exon vs cassette exons; P<2.2e-16 for Alu-derived exons vs constitutive exons; Wilcoxon rank sum test). We observed the same trend for the 3′ splice site. The median 3′ splice site score of Alu-derived exons was 6.79, significantly lower than the scores of cassette exons (7.86) and constitutive exons (8.87). In addition, Alu-derived exons had a lower density of exonic splicing regulatory elements (ESRs). We used two sets of ESRs from the studies of Goren et al [16] and Fairbrother et al [17]. For each exon, we calculated the density of ESRs as the number of nucleotides covered by ESRs divided by the total length of the exon. The average ESR density of Goren et al was 0.484 on Alu-derived exons, compared to 0.500 on cassette exons and 0.532 on constitutive exons (P = 0.04 for Alu-derived exon vs cassette exons; P = 6.5e-14 for Alu-derived exons vs constitutive exons). The same trend was observed for ESRs of Fairbrother et al: the average density was 0.144 on Alu-derived exons, which was significantly lower than the density on cassette exons (0.268) and constitutive exons (0.328).

We also found that Alu-derived exons had much higher evolutionary rates during primate evolution, compared to constitutive exons and cassette exons. Recently, the genome sequences of several non-human primates have become available. Therefore, we can study the sequence evolution of Alu-derived exons in primates after the initial Alu insertion events. To determine the evolutionary rate of different classes of exons, we analyzed the pairwise alignments of the human genome to the genomes of chimpanzee, orangutan, macaque and marmoset, which were increasingly distant from humans [18]. For exons present in both human and chimpanzee genomes, the overall nucleotide substitution rate of Alu-derived exons was 1.34%, compared to 0.73% for cassette exons and 0.52% for constitutive exons (P≤2.2e-16 in Alu vs cassette exons and Alu vs constitutive exon comparisons, Wilcoxon rank sum test). Similarly, between human and orangutan genomes, the overall nucleotide substitution rates of Alu-derived exons, cassette exons and constitutive exons were 3.69%, 1.81%, and 1.31% respectively. The same trend was also observed in pairwise comparisons of human-macaque and human-marmoset genomes (see Table 1). We also obtained similar results when we restricted our analysis to exons smaller than 250 nt (data not shown). These comparative analyses span the last ∼50 million years of primate evolution [18].

**Table 1:** The nucleotide substitution rates of three classes of exons during primate evolution.

Human VS	Exons	Number of nucleotides		
		conserved	substituted	% substituted
Chimpanzee	Alu-derived exons	59812	813	1.34
	cassette exons	670739	4925	0.73
	constitutive exons	1486944	7822	0.52
Orangutan	Alu-derived exons	51102	1957	3.69
	cassette exons	620988	11452	1.81
	constitutive exons	1371194	18245	1.31
Macaque	Alu-derived exons	37122	2801	7.02
	cassette exons	619754	21563	3.36
	constitutive exons	1428543	36227	2.47
Marmoset	Alu-derived exons	23863	3146	11.65
	cassette exons	509368	31128	5.76
	constitutive exons	1132952	50968	4.31

Taken together, these data are consistent with the hypothesis that the majority of primate-specific human exons derived from Alu elements are evolutionary intermediates without established functions [4],[6]. The high evolutionary rate of Alu-derived exons observed in primate genome alignments probably reflects the combined effect of reduced negative selection pressure on non-functional Alu exons as well as positive selection pressure on Alu exons with adaptive benefits. However, distinguishing the effect of positive selection from that of the reduced negative selection is a difficult task in general [19],[20]. Identifying the subset of Alu exonization events that have undergone positive selection using sequence-based approaches is particularly difficult for some practical reasons. Most Alu-derived exons are short (median length of the 330 exons is 121 nucleotides). They are too new to have homologous sequences from distantly related species – homologous sequences of these exons may only exist in non-human primates. Thus, for most exons the number of nucleotide differences between homologous sequences is small, which significantly decreases the power of statistical tests. Although SNP-based approaches have been applied to genome-wide scans of positive selection on the human genome [21]–[26], the regions identified by these studies are typically very large, making it a major challenge to locate the causal allele for positive selection [27]. In addition, SNP-based methods are sensitive to the temporal phases of positive selection [28], influenced by the ascertainment bias [29], and confounded by demographic factors [19], [30]–[32]. For example, the Alu-derived exon of ADAR2 (ADARB1) is a well-known case of functional exonization. This exon inserts an in-frame peptide segment into the catalytic domain of ADAR2, altering its catalytic activity [33]. Using HapMap (I+II) SNP data [21],[34], we tested for the reduction of SNP heterozygosity, the skewed allele frequency spectrum with Tajima's *D* [35] and Fay and Wu's *H* [36], and the increased population differentiation (Fst) [26],[37] (see details of the analysis in Text S1). We did not observe evidence of positive selection on this ADAR2 exon using these metrics (see Figure S1A). Similarly, SNP-based tests did not indicate evidence of positive selection for the alternative first exon of p75TNFR (see Figure S1B), the result of another well-known functional exonization event [8]. These data show the limitation of using sequence-based approaches to identify functional Alu exonization events.

A direct approach to assess the impact of individual Alu-derived exons on mRNA and protein products is to examine the splicing patterns of these exons in human tissues. Therefore, we proceeded with a large-scale splicing analysis of Alu-derived exons, using Affymetrix Exon array data of 330 exons in 11 human tissues and RT-PCR experiments of 38 exons, described in detail below.

### Affymetrix Exon Array Data on Alu-Derived Exons

To examine the splicing patterns of Alu-derived exons, we used a public Affymetrix Exon 1.0 array data set on 11 human tissues (breast, cerebellum, heart, kidney, liver, muscle, pancreas, prostate, spleen, testes, thyroid) [38], with three replicates per tissue. The Affymetrix human Exon 1.0 array is a high-density exon-tiling microarray platform designed for genome-wide analysis of pre-mRNA splicing, with over six million probes for well-annotated and predicted exons in the human genome [39],[40]. Most exons are targeted by a probeset of four perfect-match probes.

We compiled a list of 330 Exon array probesets targeting the 330 Alu-derived exons (see details in Materials and Methods). In each of the 330 probesets, we had at least three probes to infer the splicing profile of the exon, after we filtered probes showing abnormal intensities (Materials and Methods). Using a series of statistical methods that we developed for Exon array analysis [41],[42], for each probeset targeting an Alu-derived exon, we calculated the background-corrected intensities of its multiple probes and the overall expression levels of the gene in 11 tissues. These data were used to infer the splicing patterns of the exon.

### Diverse Splicing Patterns of Alu-Derived Exons in 11 Human Tissues

A large fraction of the 330 Alu-derived exons had low probe intensities in all surveyed tissues. Using a presence/absence call algorithm we developed for Exon array analysis, which compares the observed intensity of a probe to its predicted background intensity, we summarized a probeset-level Z-score for each exon in individual tissues as in [41]. A high Z-score suggests that the target exon is expressed. 174 (53%) Alu exons had a Z-score of greater than 6 in at least one tissue, including 119 (36%) exons whose Z-score was greater than 10 in at least one tissue. We also applied the same Z-score calculation to 37687 “background” probes on Exon array. These probes do not match any known genomic and transcript sequence in mammalian genomes [43], so we can use their Z-score to estimate the false positive rate of the analysis. 5% of the background probes had Z-score greater than 6 in at least one tissue, including 3% whose Z-score was greater than 10 in at least one tissue. Based on these false positive rate estimates, at the Z-score cutoff of either 10 or 6, we estimated that 33%–48% of the 330 Alu-derived exons in our study were expressed in some of the tissues. The remaining exons were not expressed at all or were expressed at very low levels in these 11 adult tissues. Of course, this is only a rough estimate, because the Z-score of individual probesets could be affected by a variety of microarray artifacts such as low probe-affinity or cross-hybridization [44],[45]. Overall, these data are consistent with the observation that most Alu-derived exons had low transcript inclusion levels in EST databases [4]. Such Alu-derived exons may represent non-functional evolutionary intermediates that are rarely incorporated in the transcripts [9]. It is also possible that some of these exons are indeed expressed in other tissues or developmental states.

Despite the low transcript abundance of many Alu-derived exons, a small fraction of exons showed highly correlated probe intensities with the overall expression levels of their corresponding genes across the surveyed tissues, suggesting stable exon inclusion. We found 19 Alu-derived exons where three probes or more correlated with gene expression levels, including the well-characterized Alu-derived exon in ADAR2 (ADARB1) that inserts an in-frame peptide segment to ADAR2's catalytic domain [5]. Detailed descriptions of these 19 exons are provided in Table 2. Among the 19 “correlated” exons, 12 were in the 5′-UTR. One exon was in 3′-UTR and one exon was part of a non-coding transcript. The remaining five exons were in coding regions, including two that introduced premature termination codons. This distribution is consistent with the hypothesis that most functional Alu exonization events do not contribute to the proteome but may play a role in regulating gene expression [46],[47]. Similar to the finding by a recent study of species-specific exons [48], we observed an excess of Alu-derived internal exons in 5′-UTR as compared to 3′-UTR. This may reflect stronger negative selection pressure against exon creation in 3′-UTR because such exons could trigger mRNA nonsense-mediated decay. The 5′-UTR Alu exons may influence the transcriptional or translational regulation of their host genes, as suggested by Goodyer and colleagues [49].

**Table 2:** RT-PCR analysis of Alu-derived exons whose Exon array probe intensities correlate with overall gene expression levels.

Gene	Cluster	Probeset	Target exon location	Target exon size (bp)	PCR Skipping (bp)	PCR inclusion (bp)	Alu type	Alu strand/mRNA	Splicing pattern	Impact on mRNA/protein	Gene name	GO processes/known features
FAM55C	2634058	2634065	chr3:102,984,195–102,984,314	120	None	250	AluJb	Antisense	Constitutive inclusion	5′UTR	Family with sequence similarity 55, member C	Unknown
NLRP1	3742783	3742834	chr17:5,377,348–5,377,428	78	None	193	ALuJb	Antisense	Constitutive inclusion	Coding with alternative 3′ splice site	NLR family, pyrin domain containing 1	ATP binding, caspase activation, apoptosis
ZNF611	3869714	3869736	chr19:57923795–57923895	101	None	204	AluJb	Sense	Constitutive inclusion	5′UTR	Zinc finger protein 611	Regulation of transcription
ADAL	3591365	3591369	chr15:41412801–41412924	124	None	356/456/457	AluJb	Sense	Constitutive inclusion	5′UTR	Adenosine deaminase-like	Nucleotide metabolic process, deaminase activity
RPP38	3236538	3236542	chr10:15184223–15184341	119	None	255/264	AluJb	Antisense	Constitutive inclusion, only detected in kidney and testes	5′UTR	Ribonuclease P/MRP 38 kDa subunit	tRNA processing, hydrolase activity
RSPH10B	3037100	3037137	chr7:5973475–5973594	120	None	334	AluJb	Antisense	Constitutive inclusion, only detected in testes	5′UTR	Radial spoke head 10 homolog B (Chlamydomonas)	Unknown
EFCAB5	3716259	3716293	chr17:25424227–25424342	116	197	313	AluJo	Antisense	Alternative major form, no tissue specificity detected	Coding with alternative 3′/stop codon/3′UTR	EF-hand calcium binding domain 5	Calcium ion binding
GOLGA8A	3617458	3617512	chr15:32470616–32470919	142/228/229	178	320/406/407	AluSx	Antisense	Alternative major form, no tissue specificity detected	5′UTR	Golgi autoantigen, golgin subfamily a, 8A	Golgi apparatus protein
FLJ42842	3727033	3727035	chr17:46771967–46772084	118	198	316	AluJb	Antisense	Alternative major form, no tissue specificity detected	3′UTR	Unknown	Unknown
ADARB1	3924041	3924084	chr21:45428817–45428936	120	142	262	AluJb	Antisense	Alternative major form, no tissue specificity detected	Coding region, decrease catalytic activity [33]	Adenosine deaminase, RNA-specific, B1	mRNA processing, adenosine deaminase activity
C16orf61/DC13	3701384	3701391	chr16:79571876–79571985	110	152	262	AluJo	Antisense	Alternative medium form, no tissue specificity detected	Exon in a non-coding transcript	Homo sapiens chromosome 16 open reading frame 61	Unknown
SHMT1	3748323	3748350	chr17:18204453–18204591	139	148	287	ALuJb	Antisense	Alternative medium form, no tissue specificity detected	5′UTR	Serine hydroxymethyltransferase 1 (soluble)	L-serine metabolic process, transferase activity
CLEC7A	3444009	3444018	chr12:10168953–10169039	87	103/222	190/309	AluJb	Antisense	Alternative medium form, no tissue specificity detected	Coding region with in frame pre-mature termination codon OR 3′ UTR	C-type lectin domain family 7, member A	T-cell activation, inflammatory response, MHC protein binding
MIPOL1	3532935	3532943	chr14:36758509–36758647	139	230	369/470/609	AluJo	Antisense	Alternative minor form, no tissue specificity detected	5′UTR	Mirror-image polydactyly 1	Unknown
CAMKK2	3474885	3474928	chr12:120215348–120215460	123	133	256	AluSq	Antisense	Alternative minor form, no tissue specificity detected	5′UTR	Calcium/calmodulin-dependent protein kinase kinase 2, beta	MAPKKK cascade, calmodulin-dependent protein kinase activity
CCDC53	3468225	3468250	chr12:100968097–100968199	103	147	250	AluJo	Antisense	Alternative minor form, no tissue specificity detected	Coding with premature termination codon	Coiled-coil domain containing 53	Unknown
SLFN11	3753500	3753521	chr17:30718078–30718195	118	156	253/274/324/372	AluJb	Antisense	Pancreas specific minor form, alternative major form in most tissues	5′UTR.	Schlafen family member 11	ATP binding, nucleotide binding
NOX5	3599561	3599599	chr15:67054122–67054224	103	128	231/276	AluJb	Antisense	Liver, pancreas and testes specific skipping, constitutive inclusion in other tissues	5′UTR	NADPH oxidase, EF-hand calcium binding domain 5	NADPH oxidase that generates superoxide and functions as a H+ channel in a Ca(2+)-dependent manner
B3GALNT1	2703377	2703394	chr3:162290426–162290544	119	253	372	AluSx	Antisense	Cerebellum, heart and testes specific minor form	5′UTR	beta-1,3-N-acetylgalactosaminyltransferase 1	Protein amino acid glycosylation, galactosyltransferase activity

Several types of splicing patterns could explain the observed correlation between probe intensities and estimated gene expression levels. These “correlated” exons could be constitutively spliced, or alternatively spliced at similar levels across tissues, or alternatively spliced but with certain variations in exon inclusion levels from tissue to tissue. However, we could not distinguish these situations based on Exon array data alone, since uncertainties in microarray probe affinity [44] prevent estimations of the absolute transcript abundance of individual exons.

**Figure 1:**
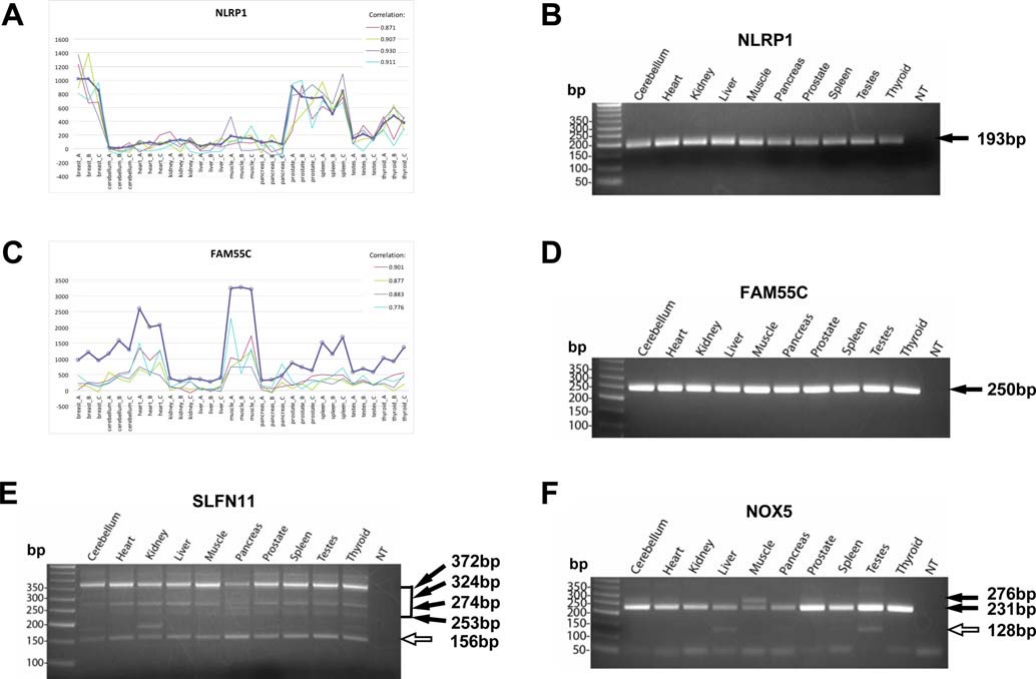
Examples of “Correlated” Exons analyzed by Exon Array analysis, semi-quantitative RT-PCR and sequencing. A. Exon array analysis of NLRP1. B. RT-PCR analysis of Alu-derived exon in NLRP1. C. Exon array analysis of FAM55C. D. RT-PCR analysis of Alu-derived exon in FAM55C. E. RT-PCR analysis of Alu-derived exon in SLFN11. F. RT-PCR analysis of Alu-derived exon in NOX5. In Exon Array analysis, the bold line represents the overall gene expression levels across all 11 tissues, each with 3 replicates; each of the fine lines represents the background corrected intensities of a probe targeting the Alu-derived exon. The Pearson correlation coefficient of the individual probe's intensities with the estimated gene expression levels in 11 tissues is shown at the top right corner of each graph. In each gel figure, solid arrows show sequencing analysis confirmed Alu exon inclusion forms. Hollow arrows show sequencing analysis confirmed Alu exon skipping forms.

To uncover the exact splicing patterns of the “correlated” exons we analyzed all 19 exons by RT-PCR, using RNAs from all available tissues surveyed by Exon array (purchased from Clontech, Mountain View, CA) except breast tissue. For each exon, we designed RT-PCR primers targeting its flanking constitutive exons. The identities of all PCR products close to the expected sizes of exon inclusion or skipping forms were further confirmed by sequencing (Materials and Methods). We discovered three major categories of splicing patterns in these 19 exons (Table 2). Six exons (in FAM55C, NLRP1, ZNF611, ADAL, RPP38, RSPH10B) were constitutively spliced. For example, the four probes of an Alu-derived exon in NLRP1 had a minimal correlation of 0.86 with the expression levels of NLRP1 in the Exon array data (Figure 1A). Our RT-PCR analysis showed a single isoform corresponding to the exon inclusion form in all surveyed tissues (Figure 1B). In FAM55C, an Alu-derived exon was shown previously to be included in the only isoform product in a human neuroblastoma cell line [13]. We found all four probes of this FAM55C exon had a minimal correlation of 0.78 with the overall gene expression levels (Figure 1C). Our RT-PCR experiments showed that this exon was constitutively spliced (Figure 1D). In another three tested genes (SLFN11, NOX5, B3GALNT1), the Alu-derived exons were alternatively spliced, but the transcript inclusion levels varied in individual tissues. For example, the SLFN11 exon was included in the major transcript product in most tissues but appeared as the minor form in pancreas. We observed Alu exon inclusion isoforms of varying lengths that resulted from alternative splice site usages of the Alu-derived exon and its upstream alternative exon (Figure 1E). In NOX5, a single exon-inclusion isoform was detected in most tissues, but an additional exon-skipping isoform was detected in liver, pancreas and testes (Figure 1F). In the remaining 10 tested genes, the exons were alternatively spliced with varying levels of transcript inclusion, but no exon showed evidence of tissue-specificity in our semi-quantitative RT-PCR analyses (see Table 2 and Figure S2).

We also conducted RT-PCR analyses of 11 “uncorrelated” exons (Table S1). The lack of correlation between probe intensities of an exon and overall gene expression levels can be due to a number of reasons. If the target Alu-derived exon has very low transcript inclusion levels, or if the probes have poor binding affinity to the target exon, the intensities of the microarray probes could be largely saturated by microarray noise, resulting in poor correlation with the overall gene expression levels. It is also possible that the correlation pattern of a highly expressed Alu-derived exon is obscured due to microarrray artifact (such as cross-hybridization) in a subset of samples. Thus, by analyzing “uncorrelated” exons, especially those with high probeset-level Z-scores in individual tissues, we may discover additional Alu-derived exons with high transcript inclusion levels. Indeed, among six RT-PCR tested “uncorrelated” exons whose probeset-level Z-score was greater than 7 in at least three tissues, we found two constitutive exons, three exons with medium to high transcript inclusion levels, as well as one exon in the minor transcript isoform (see Table S1 and Figure S3). By contrast, among five exons whose probeset-level Z-score was smaller than 3 in all 11 tissues (suggesting weak exon inclusion), four exons had very weak exon-inclusion transcripts in all surveyed tissues. The exon in FAM124B had medium transcript inclusion levels (see Figure S3).

Taken together, our RT-PCR analysis of 19 “correlated” exons and 11 “uncorrelated” exons indicates that a subset of Alu-derived exons have acquired strong splicing signals, so that they are included in the transcript products at high levels. Moreover, while prior EST-based analyses suggested all Alu-derived exons to be alternatively spliced [4], we provide experimental evidence that some Alu-derived exons are constitutively spliced in a broad range of normal human tissues.

### Discovery of Tissue-Specific Alu-Exons

**Figure 2:**
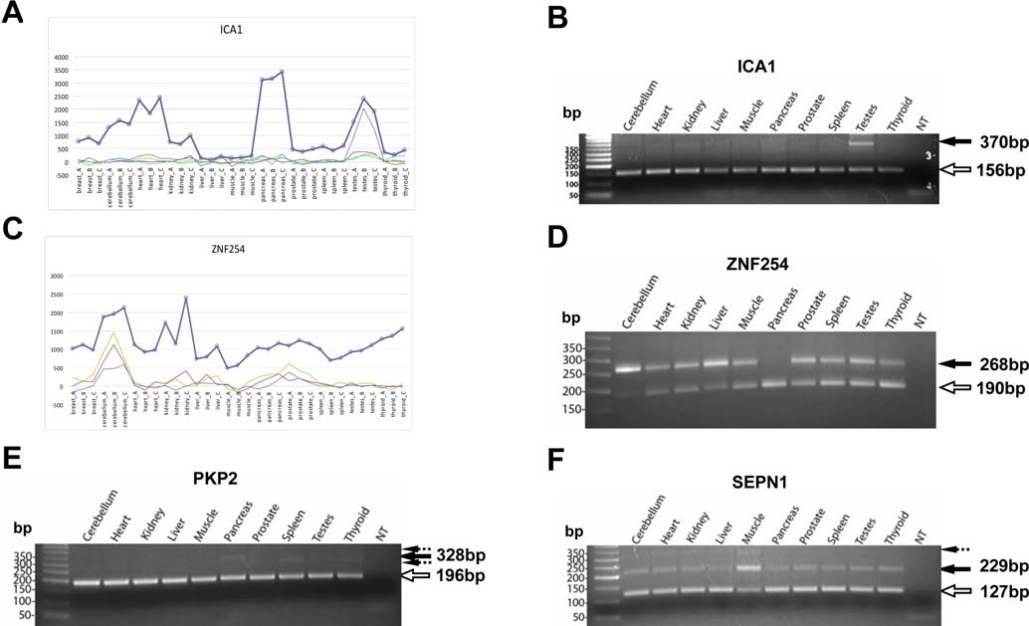
Examples of tissue-specific Alu-derived exons analyzed by Exon Array analysis, semi-quantitative RT-PCR and sequencing. A. Exon array analysis of ICA1 indicates a testes specific inclusion of Alu-derived exon. B. RT-PCR analysis of Alu-derived exon in ICA1. C. Exon array analysis of ZNF254 indicates a cerebellum specific inclusion of Alu-derived exon. D. RT-PCR analysis of Alu-derived exon in ZNF254. E. RT-PCR analysis of Alu-derived exon in PKP2. F. RT-PCR analysis of Alu-derived exon in SEPN1. In Exon Array analysis, the bold line represents the overall gene expression levels across all 11 tissues, each with 3 replicates; each of the fine lines represents the background corrected intensities of a probe targeting the Alu-derived exon. In each gel figure, solid arrows show sequencing analysis confirmed Alu exon inclusion forms. Hollow arrows show sequencing analysis confirmed Alu exon skipping forms. Dashed arrows show sequencing analysis confirmed non-specific PCR products.

Our analysis of the “correlated” Alu exons revealed that some exons had varying transcript inclusion levels in different tissues. It is possible that exons with strong tissue-specific splicing patterns do not have highly correlated intensities with the overall gene expression levels, and were missed by the above analysis. Therefore, we combined computational analysis and manual inspection of Exon array data to specifically search for tissue-specific exons (see Materials and Methods). We selected three exons (in ICA1, ZNF254, FAM79B/TPRG1) that appeared to exhibit strong tissue-specific splicing patterns for RT-PCR. We also selected five other Alu-derived exons with prior experimental evidence for exon inclusion in at least one tissue or cell line [9],[12],[50],[51], regardless of whether reliable Exon array probes existed for these exons (Table 3). Our RT-PCR experiments detected four exons with tissue-specific splicing patterns (also see Figure S4 for the other four exons with no tissue-specificity). In ICA1, the Exon array data suggested testes-specific exon inclusion (Figure 2A). The RT-PCR analysis detected a strong band corresponding to the exon inclusion form specifically in the testes (Figure 2B). In ZNF254, the RT-PCR analysis indicated strong exon inclusion in cerebellum, which was consistent with the Exon array profile (Figure 2C–D). We also found that this exon was almost completely skipped in pancreas, although this pattern was not observed in the Exon array data. In PKP2, the exon inclusion form was shown to be the minor isoform in HT29, a colon cancer cell line [9]. Our RT-PCR result showed that this exon was skipped in all other surveyed tissues but was included in the minor transcript product in the pancreas (Figure 2E).

**Table 3:** Detection of tissue-specific splicing in candidate Alu-derived exons selected from Exon array analysis and published literature.

Gene	Cluster	Probeset	Target exon location	Target exon size (bp)	PCR Skipping (bp)	PCR inclusion (bp)	Alu type	Alu strand/mRNA	Observed Splicing pattern	Impact on mRNA/protein	Prior evidence of Exon Splicing	Gene name	GO processes/known features
ICA1	3038065	3038156	chr7:8233793–8234006	214	156	370	AluJo	Sense	Testes specific inclusion	Pre-mature stop or alternative start	Testes-specific inclusion based on Exon array data	Islet cell autoantigen 1	Neurotransmitter transport, autoantigen in insulin-dependent diabetes mellitus and primary Sjogren's syndrome
ZNF254	3827427	3827448	Chr19: 24023579–24023656	78	190	268	AluJb	Antisense	Cerebellum specific major form, pancreas specific skipping, alternative medium form in other tissues	5′UTR	Cerebellum-specifc inclusion based on Exon array data	Zinc finger protein 254	DNA binding, negative regulation of transcription from RNA polymerase II promoter
SEPN1	2326126	2326133	chr1:26001094–26001195	102	127	229	AluJb	Antisense	Muscle specific major form, alternative minor form in most tissues	Coding sequence, but no protein detection in previous report	Alternative minor form predicted from ESTs and RT-PCR [50]	Selenoprotein N, 1	Calcium ion binding,mutations in this gene cause the classical phenotype of multiminicore disease and congenital muscular dystrophy with spinal rigidity and restrictive respiratory syndrome.
PKP2	3450234	3450257	chr12: 32887387–32887503	132	196	328	AluSg	Sense	Pancreas specific inclusion	Coding	Alternative minor form in HT29 cell line by RT-PCR [9]	Plakophilin 2	Cell-cell adhesion
RPE	2525852	2525861	chr2:210589173–210589226	54	233	287	AluJ/FRAM	Antisense	Alternative minor form, no conclusive evidence for tissue-specificity	Coding	Alternative minor form in placenta by RT-PCR [9]	Ribulose-5-phosphate-3-epimerase	Carbohydrate metabolic process, ribulose-phosphate 3-epimerase activity
SUGT1	N/A	N/A	chr13:52133611–52133706	96	98	194	AluSx	Antisense	Alternative medium form, no conclusive evidence for tissue-specificity	Coding	Uterus, pancreas, muscle-specific based on EST data [12]	Suppressor of G2 allele of SKP1	Mitosis
FAM79B (TPRG1)	2657546	2657554	chr3:190201257–190201394	138	153	291	AluJo	Antisense	Alternative major form, no conclusive evidence for tissue-specificity	5′UTR	Kidney-specific inclusion based on Exon array data	Tumor protein p63 regulated 1	Unknown
BCL2L13	3936256	3936278	chr22:16547375–16547472	98	152	None	AluSp	Antisense	No detectable inclusion in any tested tissue	Coding with premature stop codon	Alternative minor form in heart, HeLa cell line, lymphocyte by RT-PCR [51]	BCL2-like 13 (apoptosis facilitator)	Caspase activation,induction of apoptosis

Some tissue-specific Alu-derived exons have interesting functional implications. For example, SEPN1 encodes selenoprotein N, 1, which is expressed in skeletal muscle and has been suggested to play a role in protection against oxidant damage [50]. Mutations in SEPN1 were linked to a form of congenital muscular dystrophy [50]. SEPN1 is expressed as two alternatively spliced isoforms. The full-length isoform contains an Alu-derived exon, which is predicted to be the minor isoform based on EST data. The Alu-derived exon contains a second in-frame TGA selenocysteine residue. However, the protein product corresponding to the exon inclusion isoform was not detected by Western blot in the HeLa cell [52]. Our RT-PCR result indicated a strong muscle-specific increase in the inclusion level of this Alu-derived exon (Figure 2F). It will be interesting to investigate whether this splicing pattern represents a mechanism for modulating SEPN1 activity in muscle.

**Figure 3:**
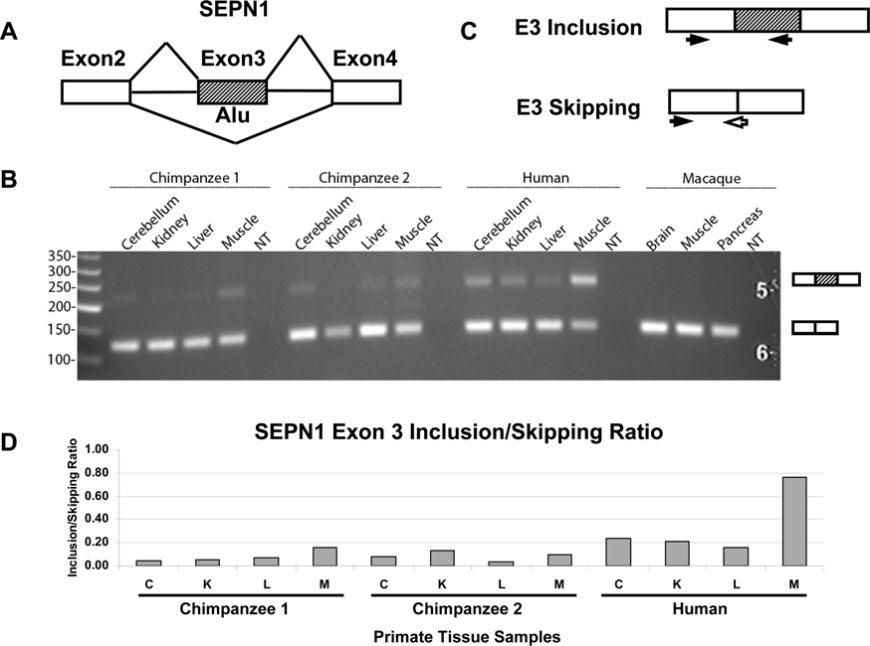
Evolution of SEPN1 Alu-exon splicing in primates. A. The splicing pattern of SEPN1 Alu-derived exon. B. RT-PCR analysis of the SEPN1 Alu-derived exon in human, chimpanzee and macaque tissues. The RT-PCR primer was designed from the upstream and downstream constitutive exon on the human gene and matched perfectly to chimpanzee and macaque transcripts. C. Realtime qPCR primers that specifically amplify exon inclusion and skipping forms. The reverse PCR primer for the skipping form was designed from the junction of upstream and downstream constitutive exons. These PCR primers perfectly matched both human and chimpanzee transcripts. D. The ratio of exon inclusion/skipping in human tissues and tissues of two chimpanzees estimated by realtime qPCR. The SEPN1 exon showed strong exon inclusion in human muscle but not in chimpanzee muscle. C, cerebellum; K, kidney; L, liver; M, muscle.

To further elucidate the evolution of this muscle-specific Alu exon in SEPN1, we obtained matching macaque and chimpanzee tissues and analyzed the splicing pattern of this exon in primate tissues using semi-quantitative RT-PCR as well as realtime quantitative PCR (see Materials and Methods). RT-PCR analysis of this exon in macaque tissues showed no exon inclusion (see Figure 3B), consistent with the fact that this Alu exon was absent from the corresponding SEPN1 region in the rhesus macaque genome. In chimpanzees, both exon inclusion and skipping forms were produced, but the exon inclusion levels were significantly lower compared to human tissues based on the RT-PCR gel pictures (Figure 3B). The splicing difference of this SEPN1 exon between humans and chimpanzees was further confirmed by realtime qPCR using isoform-specific primers (Figure 3C–D). These data depict the evolutionary history during the creation of an Alu-derived primate-specific exon and the establishment of its tissue-specific splicing pattern. Our results suggest that the strong transcript inclusion and muscle-specificity of the human SEPN1 exon was acquired after the divergence of humans and chimpanzees.

### Characteristics of Alu-Derived Exons with Substantial Transcript Inclusion Levels

**Figure 4:**
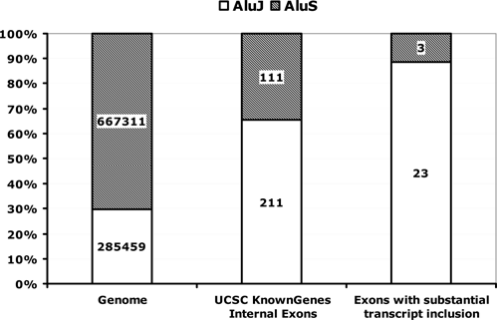
Most Alu exons with substantial transcript inclusion levels are derived from ancient Alu elements in the human genome. Plotted here are distributions of AluJ Class and AluS Class in the human genome, in Alu-derived internal exons, and in Alu-derived exons with substantial transcript inclusion levels based on our RT-PCR results. AluJ class is indicated by white column; AluS class is indicated by hatched column.

In this study, we conducted RT-PCR analysis of 38 Alu-derived exons in 10 human tissues. 26 of the 38 exons had at least medium inclusion levels in certain tissues. These exons are in genes from a wide range of functional categories (see the complete list in Table S2). Analyses of these 26 exons revealed several interesting characteristics. 23 of the 26 exons were derived from the antisense strand of Alu elements, among which 14 were from the right arm of the antisense Alu (see Figure S5), consistent with a recent report that the right arm of Alu antisense strand is a hotspot for exonization [53]. Moreover, of these 26 exons, 23 were from AluJ class and 3 were from the AluS class. By contrast, in the total set of Alu-derived exons in our study, 211 were from AluJ and 111 were from AluS, a 4-fold shift in the ratio of AluJ to AluS (7.7 in the “substantially included” set versus 1.9 in the total set; P = 0.01, one-tailed Fisher exact test). In the human genome, AluJ is outnumbered by AluS at a ratio of 1 to 2.3 [14] (Figure 4). The similar trend was also found in the 19 “correlated” exons; 16 were from the AluJ class and 3 were from the AluS class. Taken together, these data are consistent with the fact that AluJ is the oldest Alu subclass in the human genome [54], so that exons derived from AluJ elements had more evolutionary time to accumulate nucleotide changes that strengthened exon inclusion in the transcript products.

We did not observe a significant difference in the splice site score and ESR density of the 26 substantially included Alu exons compared to other Alu-derived exons (data not shown). This could be due to the lack of statistical power. Alternatively, it may reflect the current lack of knowledge of the complete set of cis-elements that regulate splicing [55],[56]. Future experimental studies (such as mini-gene experiments) are needed to dissect the exact regulatory elements important for strong transcript inclusion and/or tissue-specific splicing of individual Alu-derived exons.

## Discussion

Our study reveals diverse splicing patterns of exonized Alu elements in the human transcriptome. Most new exons originated from Alu elements probably represent non-functional splice forms that are included in the transcripts at low frequencies [4],[6]. However, a small subset of exonization events, in particular those associated with more ancient Alu elements, could evolve strong splicing regulatory signals to become constitutive or tissue-specific, possibly driven by positive selection. The analysis of high-density exon tiling array data across a broad range of tissues provides an efficient approach to identify such exons. Considering the incomplete coverage of Exon 1.0 arrays on human transcribed regions, and the high noise in the observed intensities of probes targeting individual exons [57],[58], we expect that many constitutive or tissue-specific Alu-derived exons are missed by this study. Also, while we focus on primate-specific exons derived from Alu repeats, a recent study by Alekseyenko and colleagues identified nearly 3000 human-specific exons created by de novo substitution in intronic regions during primate evolution [59]. With improved exon microarray platforms and analysis algorithms in the future, more species-specific exons with regulatory roles are likely to be discovered.

Our data provide novel insight into the evolutionary impact of newly created exons in eukaryotic genomes. During evolution, new exons are frequently added to existing functioning genes via a variety of mechanisms, such as exonization of transposable elements, exon duplication, and de novo exonization from intronic regions [6]. Modrek and Lee found that the birth of new exons was strongly coupled with widespread occurrence of alternative splicing in eukaryotic genes [60]. Through pairwise comparisons of human and rodent genomes, they showed that nearly 75% of human alternatively spliced exons with low transcript inclusion levels were absent from the corresponding genomic sequence of the rodent orthologs. By contrast, the number was less than 5% for constitutive exons [60]. This pattern was corroborated by subsequent analyses of exon creation events in vertebrates using multiple genome alignments [48],[59]. Based on these observations, Modrek and Lee proposed an evolutionary model that alternative splicing can facilitate the evolution of new exons – the creation of a new exon in the minor transcript isoform keeps the original gene product intact, which reduces the negative selection pressure against the new exon, allowing it to evolve towards an adaptive function [10],[60]. On the other hand, this evolutionary model also predicts that the vast majority of new exons found by comparative genomics analyses are non-functional evolutionary intermediates. In fact, most previous genomic studies have focused on the low transcript inclusion levels of new exons [4],[6],[48],[59],[60]. It is unclear to what extent new exons could have produced functional and regulatory novelties. In this study, based on a large-scale splicing analysis of human tissues, we show that a number of primate-specific exons derived from Alu retrotransposons have a major impact on their genes' mRNA/protein products in a ubiquitous or tissue-specific manner. In SEPN1, the strong transcript inclusion and muscle-specificity of the Alu derived exon represents a human-specific splicing change after the divergence of humans and chimpanzees. These data suggest that some new exons may contribute to species-specific differences between humans and non-human primates.

Our study has discovered a large list of Alu-derived exons with substantial transcript inclusion levels. This exon list can be valuable for a variety of further investigations. These exons provide candidates for detailed mechanistic analyses and can be used to characterize the splicing regulatory mechanisms of Alu-derived exons. If suitable tissue samples from closely or distantly related primate species are available, it will be possible to precisely reconstruct the evolutionary events preceding the emergence of constitutive or tissue-specific Alu-derived exons. Further experimental studies will be needed to elucidate the functional significance of individual exonization events (e.g. the muscle-specific inclusion of the Alu-derived exon in SEPN1).

## Materials and Methods

### Compilation of Affymetrix Exon Array Data on Alu-Derived Exons

We downloaded a public Affymetrix Exon 1.0 array data set on 11 human tissues (breast, cerebellum, heart, kidney, liver, muscle, pancreas, prostate, spleen, testes, thyroid) [38], with three replicates per tissue (http://www.affymetrix.com/support/technical/sample_data/exon_array_data.affx).

We compiled a list of Exon array probesets targeting exonized Alu elements. The locations of Alu elements in the human genome were downloaded from RepeatMasker annotation of the UCSC Genome Browser database [14]. The locations of internal exons (i.e. exons flanked by both 5′ and 3′ exons) in human genes were taken from the UCSC KnownGenes database [14]. This database combines transcript annotations from multiple sequence databases [14]. To eliminate long exonic regions likely resulting from intron retention events, we removed probesets whose probe selection regions were over 250 bp as in [61]. We then defined an exon as Alu-derived if the Alu element covered at least 25 bp of the exon and over 50% of the total length of the Exon array probe selection region. We collected 526 Exon array probesets targeting such Alu-derived exons. Since microarray probes targeting Alu repeats may cross-hybridize to off-target transcripts, we used a conservative approach to identify and remove individual probes showing abnormal intensities (see “Analysis of Exon array data” below). After probe filtering, we collected a final list of 330 Exon array probesets, with at least three reliable probes in each probeset to infer the splicing profiles of Alu-derived exons.

### Collections of Constitutive Exons and Exon Skipping Cassette Exons in the Human Genome

We collected 13103 constitutively spliced exons and 5389 exon-skipping cassette exons in the human genome, after applying stringent filtering criteria to exons in the Alternative Splicing Annotation Project 2 (ASAP2) database [62]. ASAP2 determined the splicing patterns of human exons based on the analysis of mRNA/EST sequences [62]. Constitutive exons were defined as those without any evidence of exon skipping in mRNA/EST data. To ensure that no skipping form was missed due to incomplete transcript sampling in EST databases, each constitutive exon included in our study was required to have at least 50 exon inclusion ESTs. We obtained 13103 high-confidence constitutive exons using this criterion. For exon skipping cassette exons, we collected 5389 ASAP2 exons with at least 3 inclusion ESTs and at least 3 skipping ESTs.

### Analysis of Exon Splicing Signals

For each exon, we scored its 5′ and 3′ splice sites using consensus splice site models in MAXENT [15]. For 5′ splice site, we analyzed 3 nucleotides in exons and 6 nucleotides in introns. For 3′ splice sites, we analyzed 3 nucleotides in exons and 20 nucleotides in introns. We also calculated the density of exonic splicing regulatory elements (ESRs). Two sets of elements were used separately: (i) 285 exonic splicing regulatory elements from Goren et al [16]; (ii) 238 exonic splicing enhancers from Fairbrother et al [63]. For each exon, the ESR density was calculated as the number of nucleotides covered by ESRs, divided by the total length of the exon.

### Analysis of Nucleotide Substitution Rate during Primate Evolution

To determine the nucleotide substitution rate of exons in primates, we downloaded and analyzed the UCSC pairwise genome alignments of the human genome (hg18) to the genomes of chimpanzee (panTro2), orangutan (ponAbe2), rhesus macaque (rheMac2) and marmoset (calJac1) [64]. In each pairwise alignment, we defined an exon to be conserved in a non-human primate if there was at least one homologous region that covered at least 80% of the human exon with at least 80% sequence identity. We included a conserved exon in the nucleotide substitution rate analysis if there was a single (unambiguous) homologous region in the genome alignment. For such exons, we calculated the nucleotide substitution rate between the human genome and the genome of a non-human primate as the number of conserved nucleotide within the aligned region, divided by the total length of the aligned region. The alignment analysis was performed using Pygr [65], a python bioinformatics library that provided efficient access to alignment intervals in the UCSC genome alignments.

### Analysis of Exon Array Data

Briefly, we first predicted the background intensities of individual Exon array probes, using a sequence-specific linear model [41],[66] trained from “genomic” and “anti-genomic” background probes on the Exon 1.0 array [43]. For every probe, the predicted background intensity was an estimate for the amount of non-specific hybridization to the probe. This background intensity was subtracted from the observed probe intensity before downstream analyses [41]. Second, for each gene we used a correlation-based iterative probe selection algorithm to construct robust estimates of overall gene expression levels, independent of splicing patterns of individual exons [42]. Third, since oligonucleotide probes for Alu-derived exons may be more likely to cross-hybridize than typical Exon array probes, we used two independent methods to identify and remove individual probes with abnormal probe intensities. We searched all 25mer oligonucleotide probes against all RefSeq-supported exon regions, allowing up to 3 bp mismatches. Once a potential off-target gene was found for a probe, we calculated the Pearson correlation coefficient between the probe's intensities and the off-target gene's estimated expression levels across the 11 tissues [45]. We defined a probe to be cross-hybridizing if there was an off-target gene within 3 bp mismatches, and if the computed Pearson correlation coefficient was above 0.55. Such probes were removed from further analyses. We also detected probes whose intensities were higher than 95% of all other probes for RefSeq-supported exons of the same gene in at least 3 of the 11 tissues. Such probes were regarded as outlier probes and were also removed. After probe filtering, we collected a final list of 330 Exon array probesets, with at least three reliable probes in each probeset to infer the splicing profiles of Alu-derived exons.

For each Alu-derived exon, using a presence/absence call algorithm that compares the observed intensity of a probe to its predicted background intensity, we summarized a probeset-level Z-score for exon expression in individual tissues as in [41]. We also calculated the Pearson correlation co-efficient of individual probes' intensities with the overall gene expression levels in 11 tissues (estimated from all exons of a gene, see [41],[42]). We defined a probe to be “correlated” with gene expression levels if the Pearson correlation co-efficient was above 0.6. We defined an exon to be “correlated” if it had at least three probes correlated with gene expression levels.

We used a two-step approach to identify strong tissue-specific exons, by combining computational analysis and manual inspection of Exon array data. For each probe of an exon in a tissue, we calculated a “splicing index”, defined as the background-corrected probe intensity divided by the estimated gene expression level [40]. We used a Z-score method used by Graveley and colleagues [67] to test whether the splicing index of a particular tissue was an outlier compared to other tissues . A highly positive Z-score suggests tissue-specific exon inclusion. After this initial computational screening, we manually inspected the Exon array data of potential tissue-specific exons.

### RT-PCR and Sequencing Analysis of Alu-Derived Exons in Ten Human Tissues

Total RNA samples from 10 human tissues were purchased from Clontech (Mountain View, CA). Single-pass cDNA was synthesized using High-Capacity cDNA Reverse Transcription Kit (Applied Biosystems, Foster City, CA) according to manufacturer's instructions. For each tested Alu-derived exon, we designed a pair of forward and reverse PCR primers at flanking constitutive exons using PRIMER3 [68]. Primer sequences and positions are described in Table S3. Two µg of total RNA were used for each 20 ul cDNA synthesis reaction. For each candidate Alu exonization event, 1 µl of cDNA were used for the amplification in a 25 µl PCR reaction. PCR reactions were run for 40 cycles in a Bio-Rad thermocycler with an annealing temperature of 62°C. The reaction products were resolved on 2% TAE/agarose gels. All of the candidate DNA fragments corresponding to exon inclusion and exon skipping forms were cloned for sequencing using Zero Blunt TOPO PCR Cloning Kit (Invitrogen, Carlsbad, CA).

### Real-Time qPCR Quantification of SEPN1 Exon Inclusion Level in Primate Tissues

Total RNA samples from rhesus macaque tissues (brain, skeletal muscle, pancreas) were purchased from Biochain Inc (Hayward, CA). Frozen tissue samples (cerebellum, skeletal muscle, liver, kidney) of two chimpanzees were generously provided by Southwest National Primate Research Center (San Antonio, TX). RNA was prepared using TRIzol (Invitrogen) according to the manufacturer's instructions. Single-pass cDNA was synthesized using the High Capacity cDNA Reverse Transcription Kit (Applied Biosystems, Foster City, CA). The quantitative real-time polymerase chain reaction (qRT-PCR) was performed using Power SYBR Green PCR Master Mix (Applied Biosystems, Foster City, CA). The following primers were used in qRT-PCR: SEPN1 Exon 3 skipping form: forward: 5′-GGGACAGATGGCCTTTTTCT-3′; reverse: 5′-AGTTGACCCTGTTAGCTTCTCAG-3′ ; SEPN1 Exon 3 inclusion form: forward 5′- GGAGTTCAAACCCATTGCTG -3′; reverse: 5′- AATTGAGCCAGGGAAGTTGA -3′. These qPCR primers match perfectly to their transcript targets in human and chimpanzee. Using a mathematical method described by Pfaffl [69], we calculated and presented the SEPN1 exon 3 inclusion level as a ratio to the exon 3 skipping level in each sample.

## Supporting Information

Figure S1 Negative natural log of P-values of population genetic measures around 2 Mbp regions of the Alu-exons of ADARB1 (A) and p75TNFR (B). Red vertical line indicates the position of the exon. Each point on the y-axis for these plots represents the negative natural log of P-value of the corresponding measures over the distribution of the same measures obtained from 1000 randomly selected constitutive exons (see Supplemental Methods for details). The four panels represent SNP heterozygosity (Het), Tajima's D, Fay and Wu's H, and FST. The first three are displayed for CEU and YRI populations. The green lines represent the CEU and the black lines represent YRI HapMap populations. The FST plot shows comparison between CEU and YRI populations. Note that ln(P) = 4 is equivalent to the P-value of 0.0183, thus none of the statistics around this exon is a significant outliner compared to genome-wide averages.
(0.03 MB PDF)

Figure S2 Additional “correlated” exons analyzed by Exon Array analysis, semi-quantitative RT-PCR and sequencing. A. Exon array analysis B. RT-PCR analysis of Alu-derived exons. Solid arrows show sequencing analysis confirmed Alu exon inclusion forms. Hollow arrows show sequencing analysis confirmed Alu exon skipping forms. Dashed arrows show sequencing analysis confirmed non-specific PCR products.
(0.52 MB PDF)

Figure S3 “Uncorrelated” exons analyzed by semi-quantitative RT-PCR and sequencing. RT-PCR analysis of Alu-derived exon in A. z-score<3 in 11 tissues (suggesting weak exon inclusion in all tissues) B. z-score>7 in at least 3 tissues (suggesting strong or medium exon inclusion in some tissues). Solid arrows show sequencing analysis confirmed Alu exon inclusion forms. Hollow arrows show sequencing analysis confirmed Alu exon skipping forms. Dashed arrows show sequencing analysis confirmed non-specific PCR products.
(0.29 MB PDF)

Figure S4 Four exons with no conclusive evidence for tissue-specificity by semi-quantitative RT-PCR. RT-PCR analysis of Alu-derived exon in A. RPE. B. SUGT1. C. FAM79B/TPRG1. D. BCL2L13. Solid arrows show sequencing analysis confirmed Alu exon inclusion forms. Hollow arrows show sequencing analysis confirmed Alu exon skipping forms. Dashed arrows show sequencing analysis confirmed non-specific PCR products.
(0.13 MB PDF)

Figure S5 Schematic diagram of the location and orientation of the Alu-derived exons with respect to the Alu elements. Filled black box represents Alu-derived exon. Empty box represents the full-length Alu element as annotated by UCSC Genome Browser. The orientation of ‘ALU’ in the empty box represents the orientation of the exon with respect to the Alu element (sense or antisense).
(0.01 MB PDF)

Table S1 RT-PCR analysis of Alu-derived exons whose Exon array probe intensities are uncorrelated with overall gene expression levels.
(0.02 MB PDF)

Table S2 Substantially included Alu-derived exons detected by RT-PCR analysis.
(0.02 MB PDF)

Table S3 RT-PCR primers and PCR product sizes of all tested Alu-derived exons.
(0.06 MB PDF)

Text S1 Supplemental methods. (Exon-centered genomic scan for positive selection in ADARB1 and p75TNFR).
(0.10 MB PDF)
