# Supplementary figures and images for: Diverse Splicing Patterns of Exonized Alu Elements in Human Tissues
Source: PLoS Genet. 2008 Oct 17;4(10):e1000225. doi: 10.1371/journal.pgen.1000225 (PMC2562518; doi:10.1371/journal.pgen.1000225)

A.

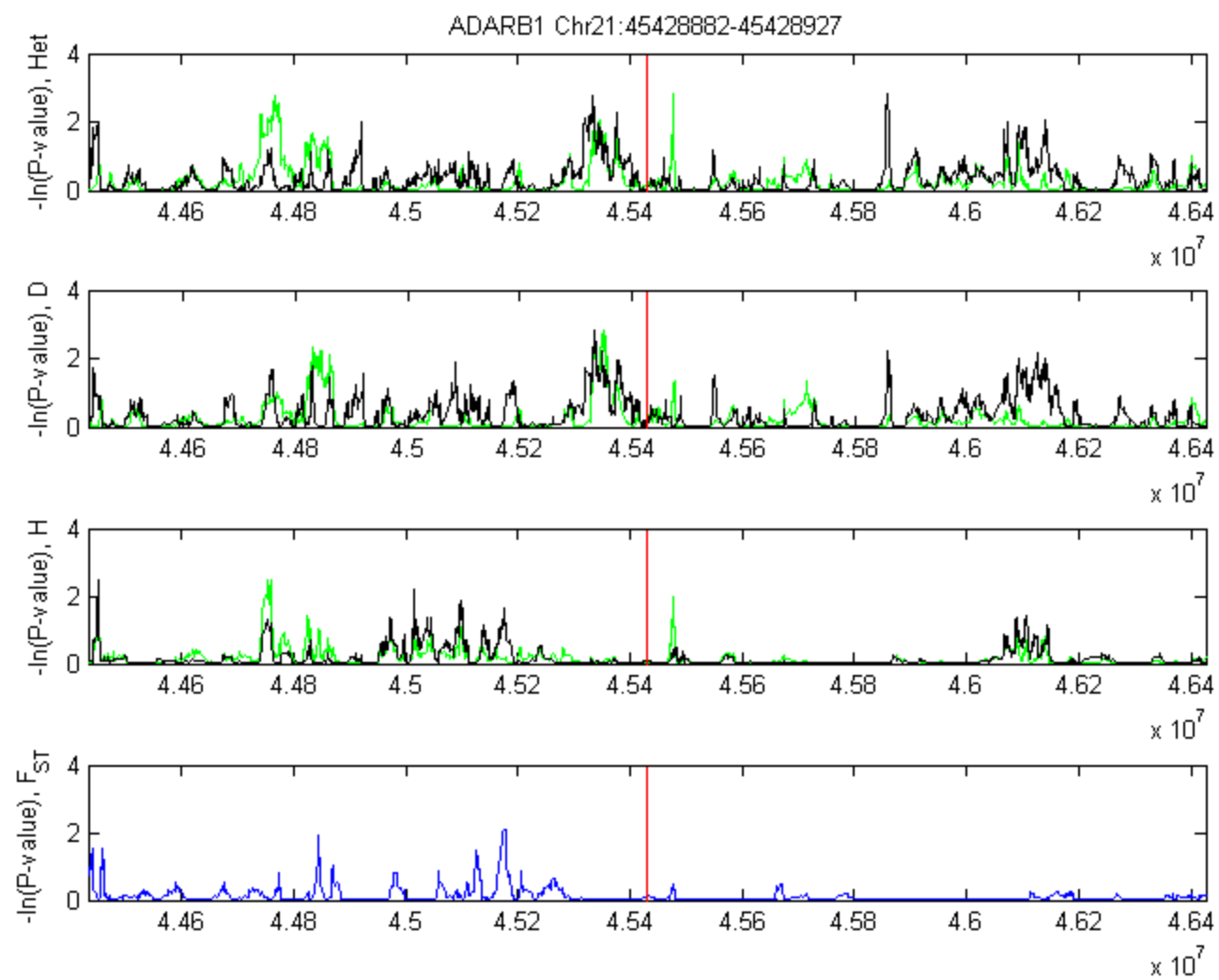

**B.**

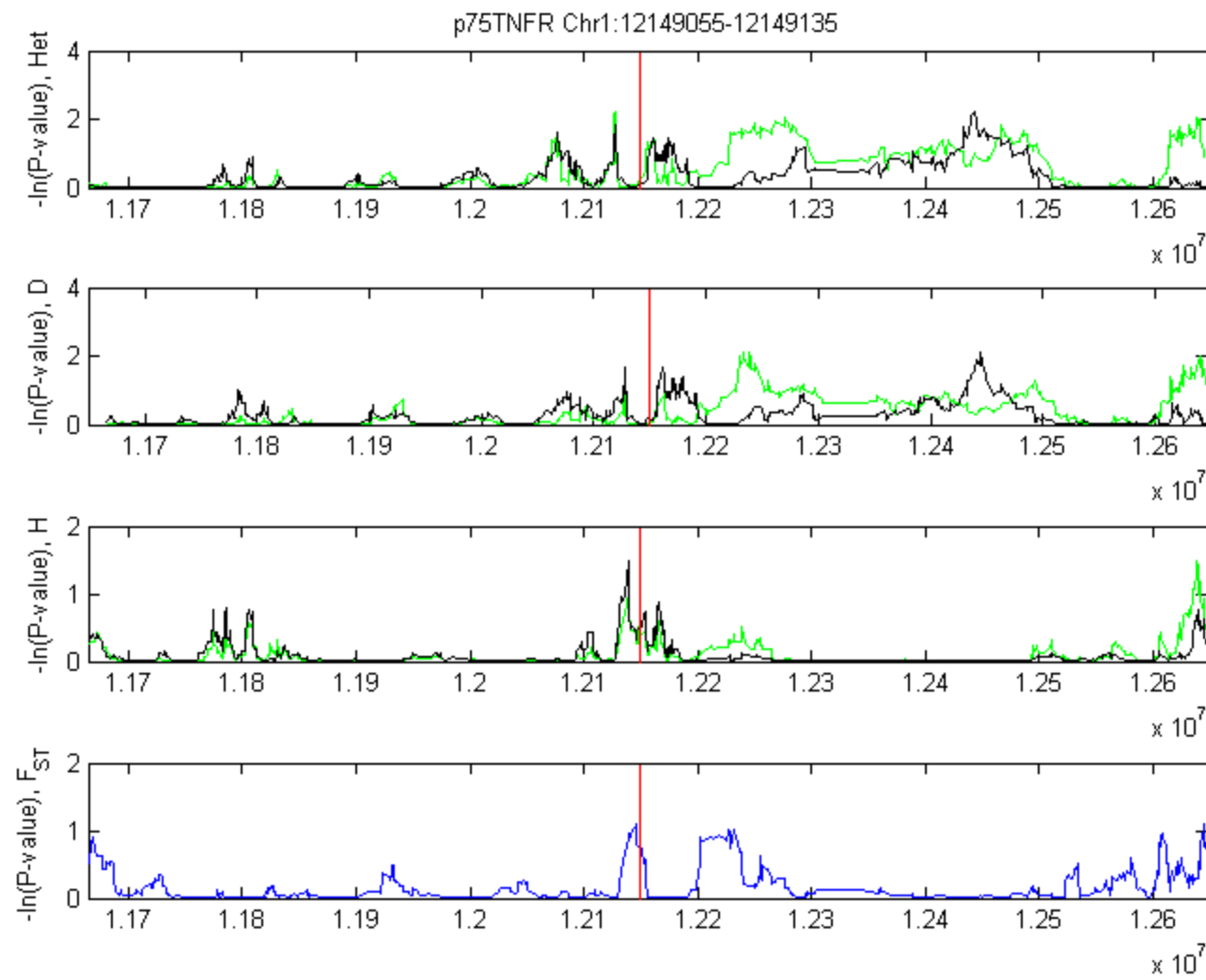

Supplement: Figure S1 — Negative natural log of P-values of population genetic measures around 2 Mbp regions of the Alu-exons of ADARB1 (A) and p75TNFR (B). Red vertical line indicates the position of the exon. Each point on the y-axis for these plots represents the negative natural log of P-value of the corresponding measures over the distribution of the same measures obtained from 1000 randomly selected constitutive exons (see Supplemental Methods for details). The four panels represent SNP heterozygosity (Het), Tajima's D, Fay and Wu's H, and FST. The first three are displayed for CEU and YRI populations. The green lines represent the CEU and the black lines represent YRI HapMap populations. The FST plot shows comparison between CEU and YRI populations. Note that ln(P) = 4 is equivalent to the P-value of 0.0183, thus none of the statistics around this exon is a significant outliner compared to genome-wide averages. (0.03 MB PDF) [file pgen.1000225.s001.pdf]

**A.**

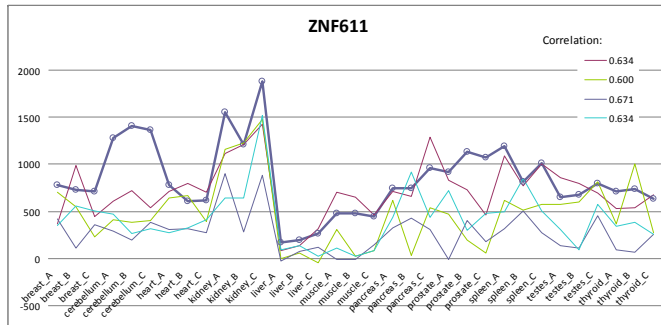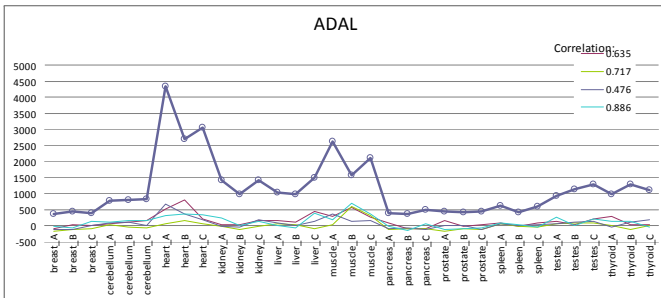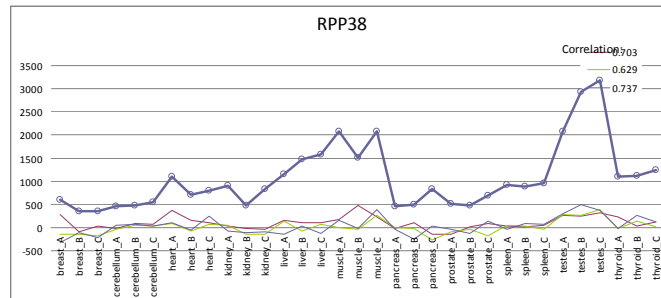

**B.**

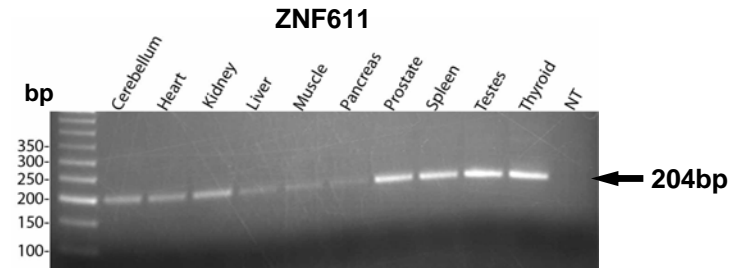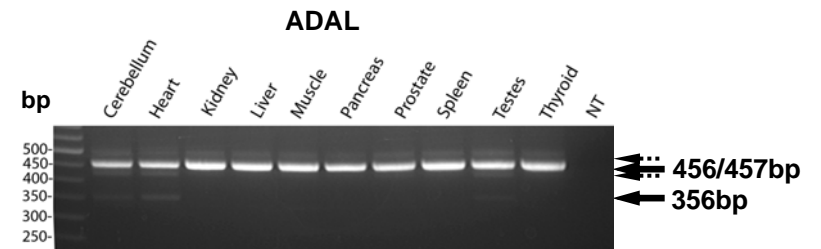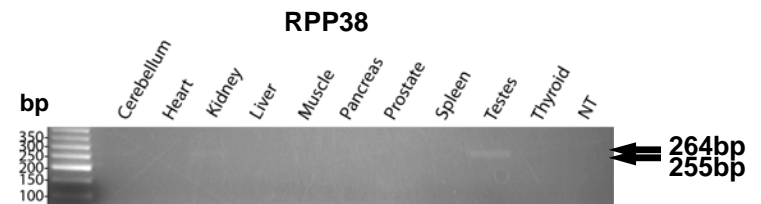

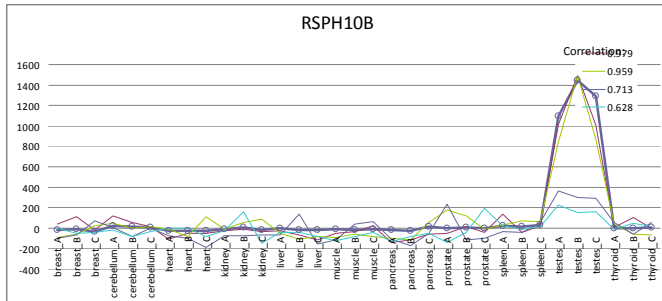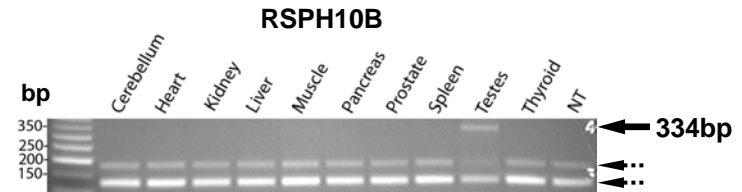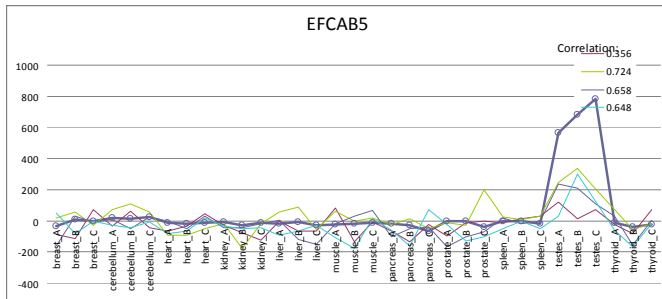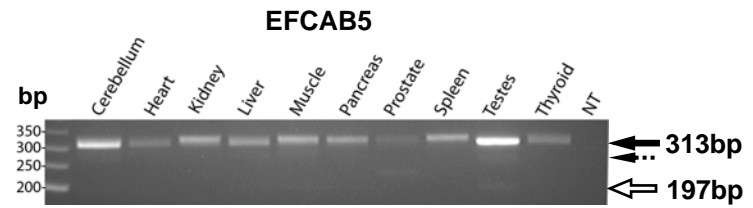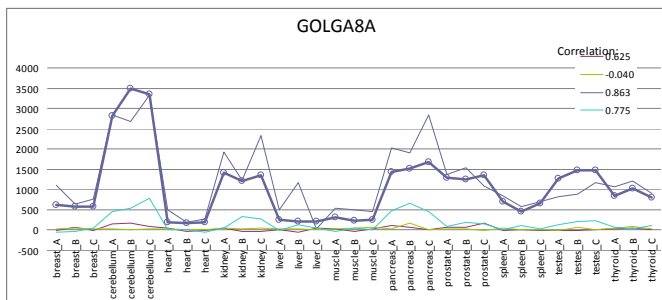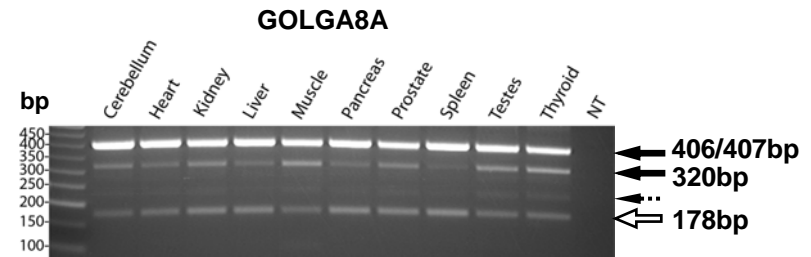

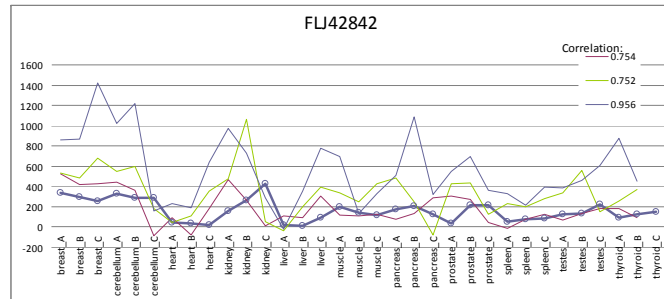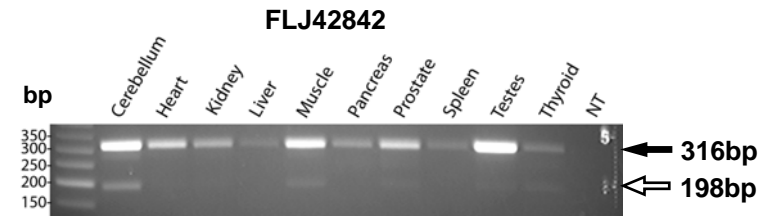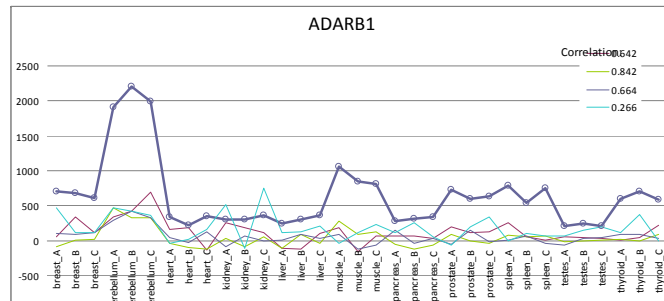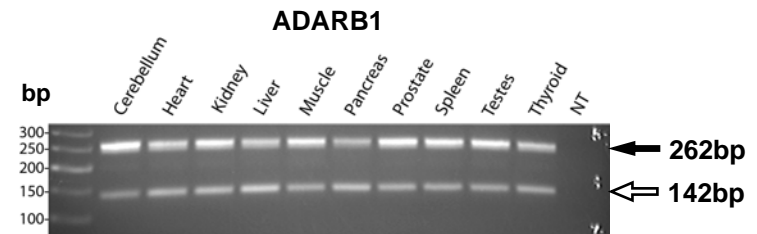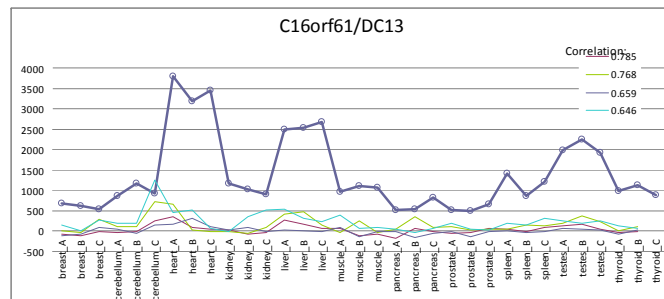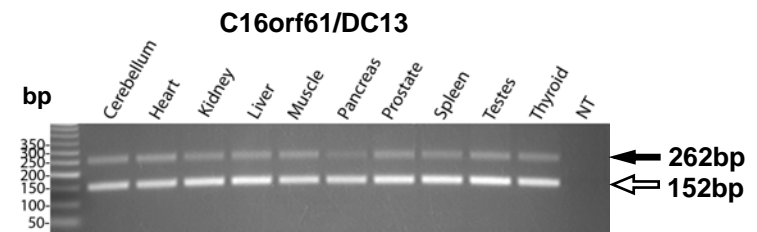

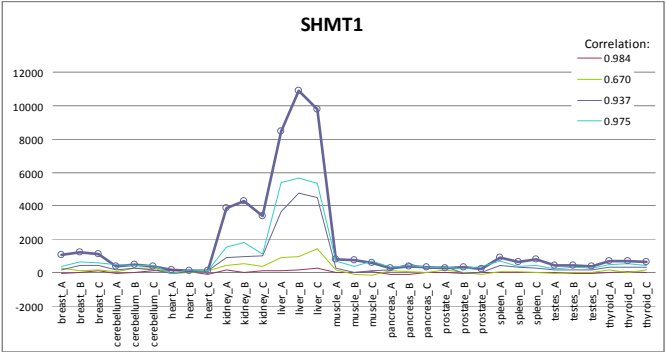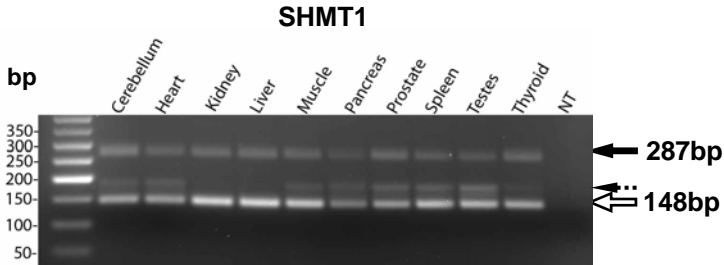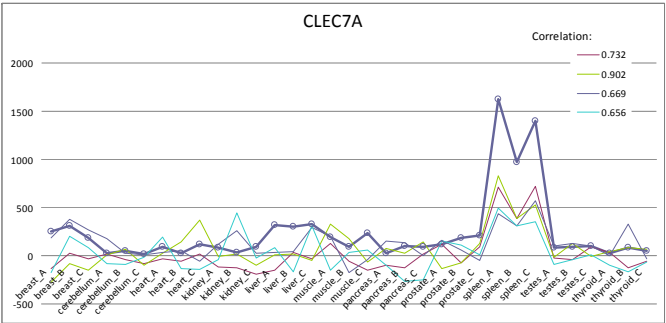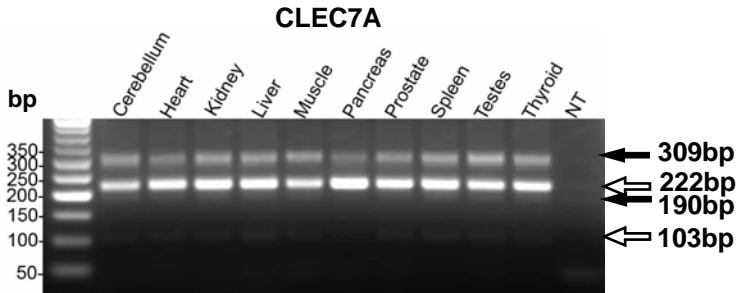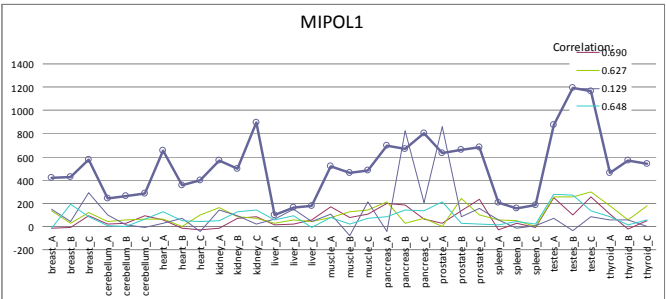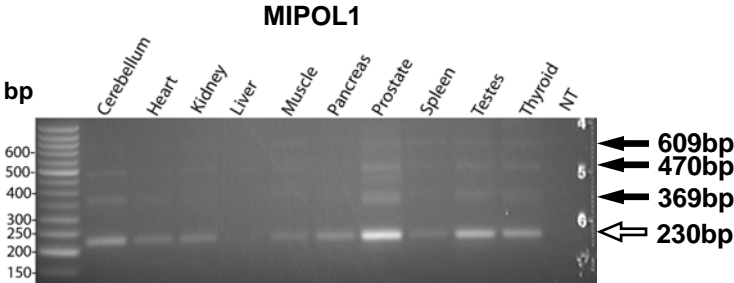

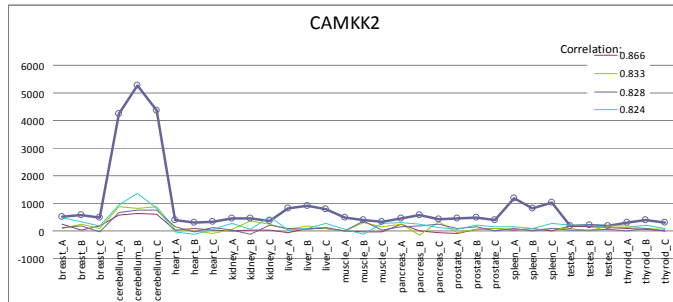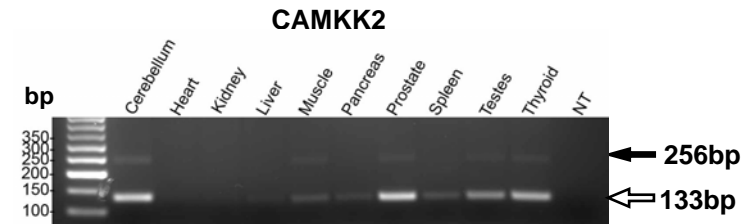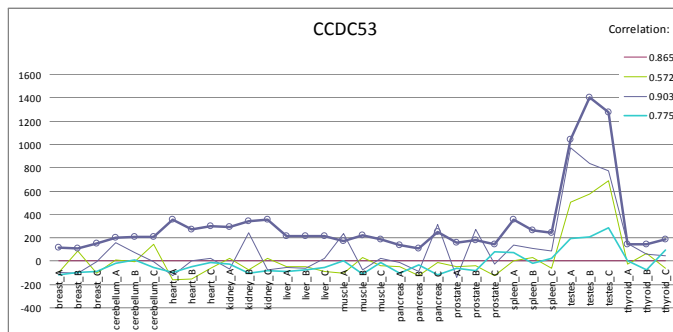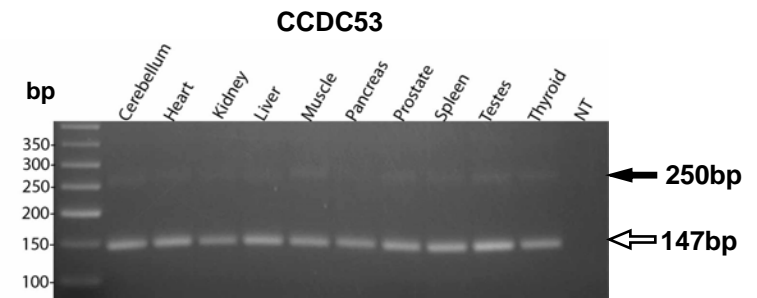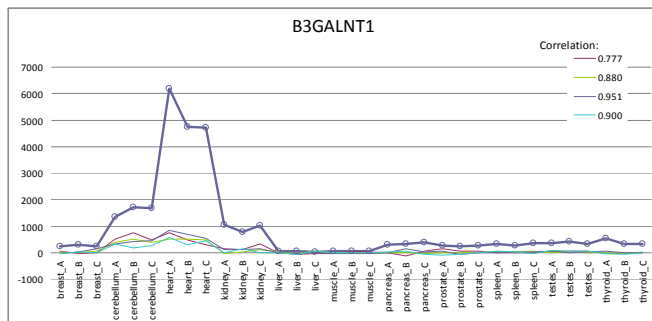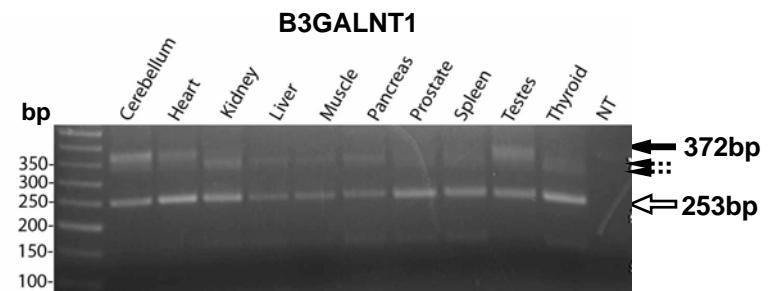

Supplement: Figure S2 — Additional “correlated” exons analyzed by Exon Array analysis, semi-quantitative RT-PCR and sequencing. A. Exon array analysis B. RT-PCR analysis of Alu-derived exons. Solid arrows show sequencing analysis confirmed Alu exon inclusion forms. Hollow arrows show sequencing analysis confirmed Alu exon skipping forms. Dashed arrows show sequencing analysis confirmed non-specific PCR products. (0.52 MB PDF) [file pgen.1000225.s002.pdf]

**A.**

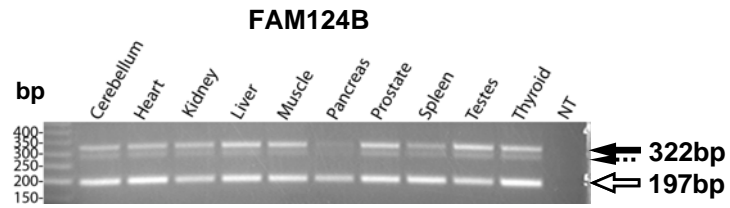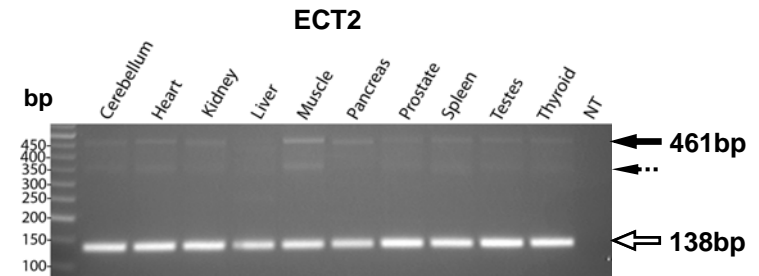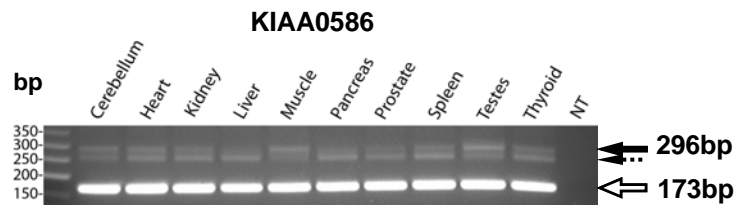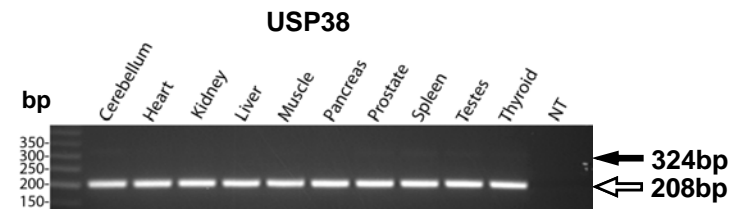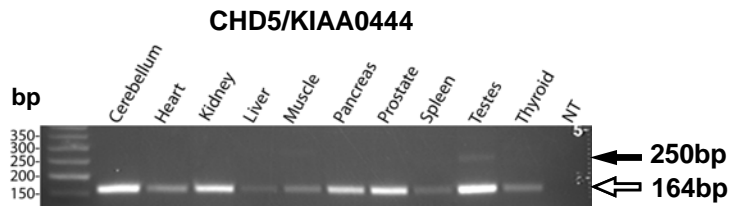

**B.**

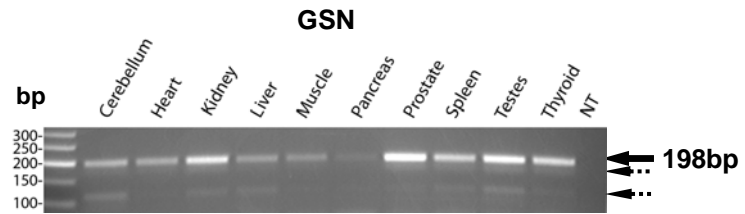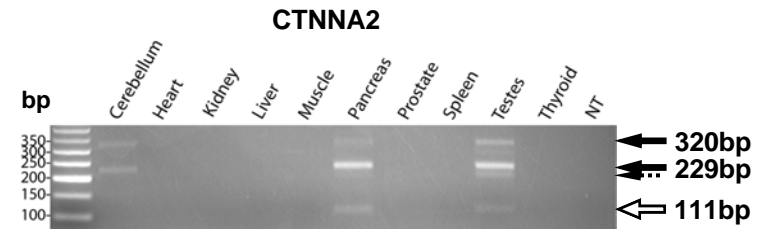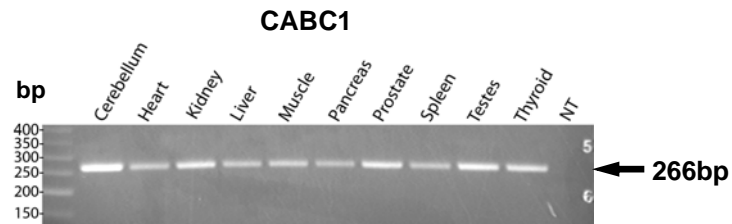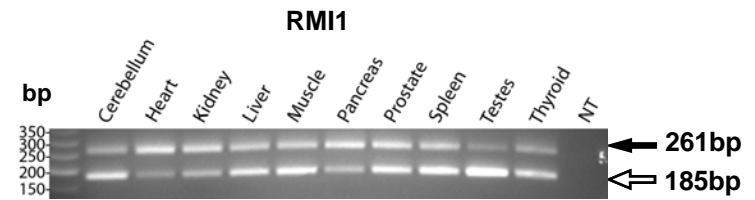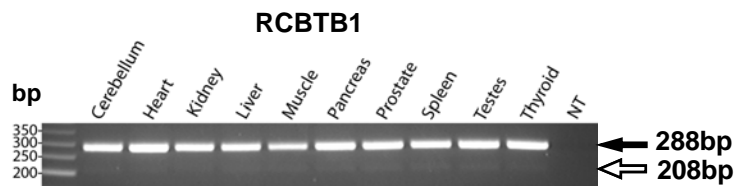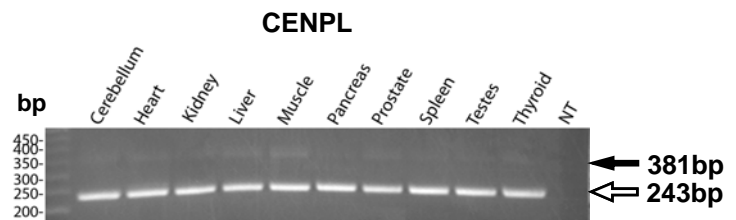

Supplement: Figure S3 — “Uncorrelated” exons analyzed by semi-quantitative RT-PCR and sequencing. RT-PCR analysis of Alu-derived exon in A. z-score<3 in 11 tissues (suggesting weak exon inclusion in all tissues) B. z-score>7 in at least 3 tissues (suggesting strong or medium exon inclusion in some tissues). Solid arrows show sequencing analysis confirmed Alu exon inclusion forms. Hollow arrows show sequencing analysis confirmed Alu exon skipping forms. Dashed arrows show sequencing analysis confirmed non-specific PCR products. (0.29 MB PDF) [file pgen.1000225.s003.pdf]

**A.**

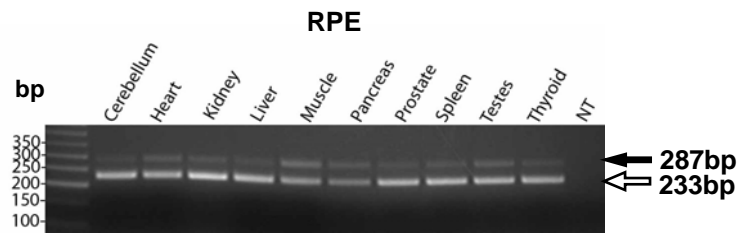

**B.**

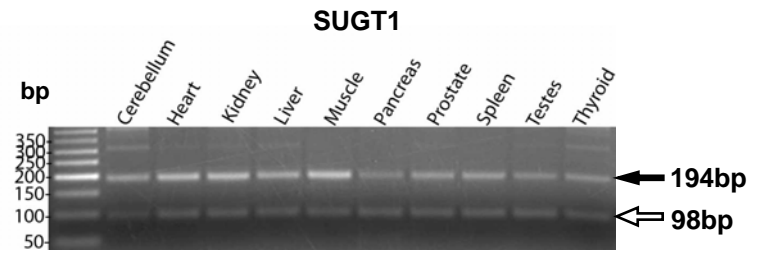

**C.**

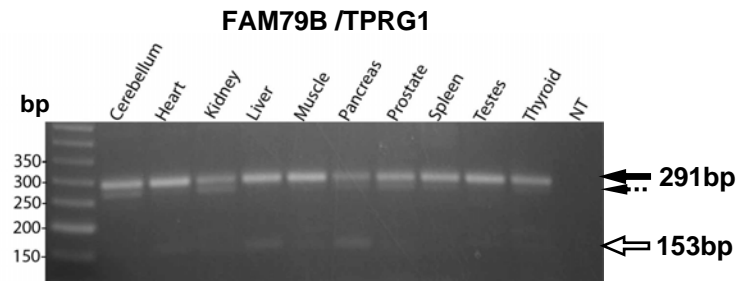

**D.**

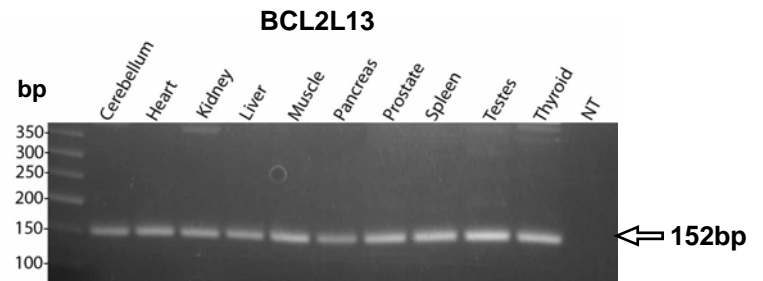

Supplement: Figure S4 — Four exons with no conclusive evidence for tissue-specificity by semi-quantitative RT-PCR. RT-PCR analysis of Alu-derived exon in A. RPE. B. SUGT1. C. FAM79B/TPRG1. D. BCL2L13. Solid arrows show sequencing analysis confirmed Alu exon inclusion forms. Hollow arrows show sequencing analysis confirmed Alu exon skipping forms. Dashed arrows show sequencing analysis confirmed non-specific PCR products. (0.13 MB PDF) [file pgen.1000225.s004.pdf]
