# Supplementary material for: Diverse Splicing Patterns of Exonized Alu Elements in Human Tissues
Source: PLoS Genet. 2008 Oct 17;4(10):e1000225. doi: 10.1371/journal.pgen.1000225 (PMC2562518; doi:10.1371/journal.pgen.1000225)

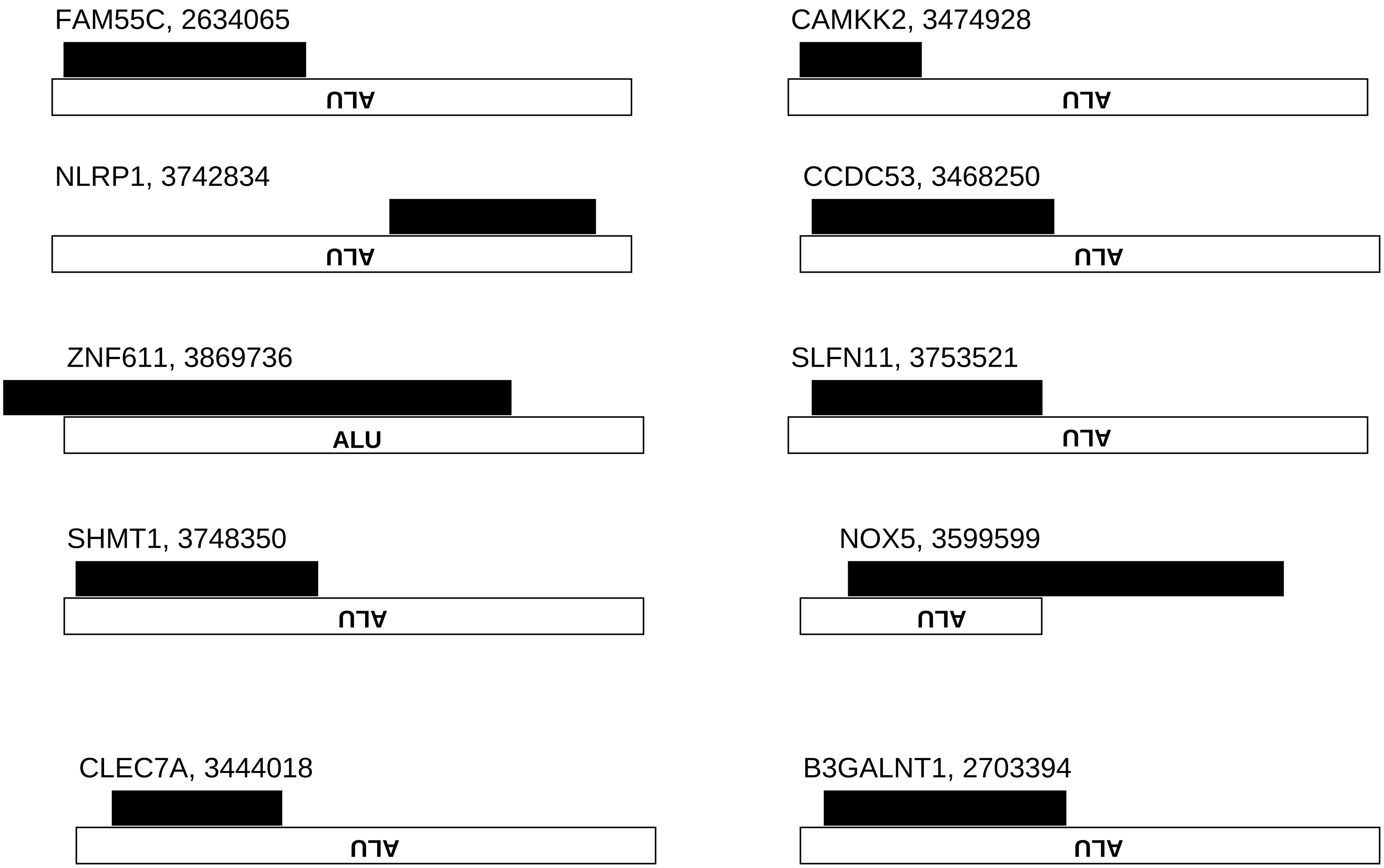
FAM55C, 2634065
ALU
CAMKK2, 3474928
ALU
NLRP1, 3742834
ALU
CCDC53, 3468250
ALU
ZNF611, 3869736
ALU
SLFN11, 3753521
ALU
SHMT1, 3748350
ALU
NOX5, 3599599
ALU
CLEC7A, 3444018
ALU
B3GALNT1, 2703394
ALU

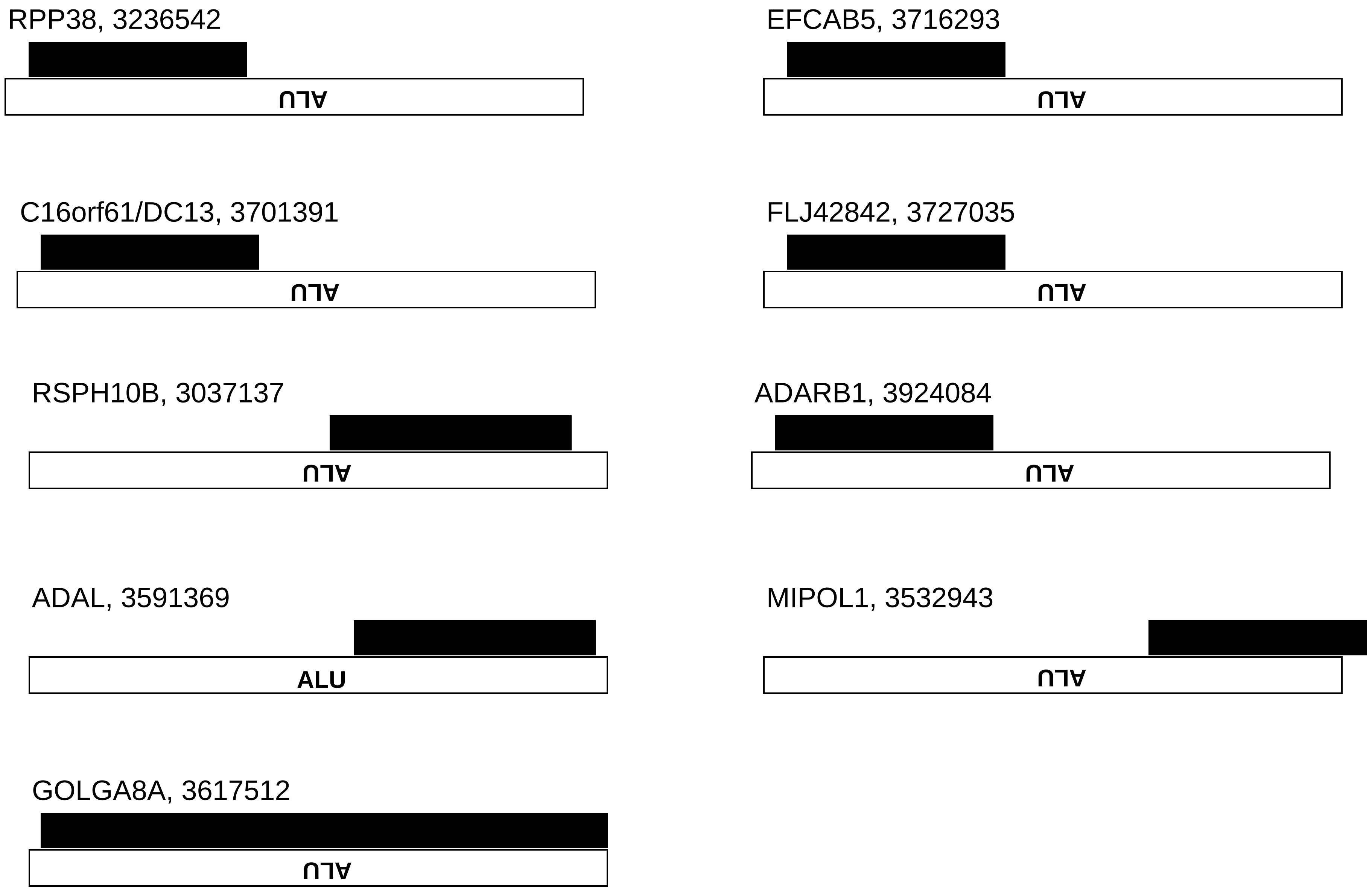
RPP38, 3236542
ALU
EFCAB5, 3716293
ALU
C16orf61/DC13, 3701391
ALU
FLJ42842, 3727035
ALU
RSPH10B, 3037137
ALU
ADARB1, 3924084
ALU
ADAL, 3591369
ALU
MIPOL1, 3532943
ALU
GOLGA8A, 3617512
ALU

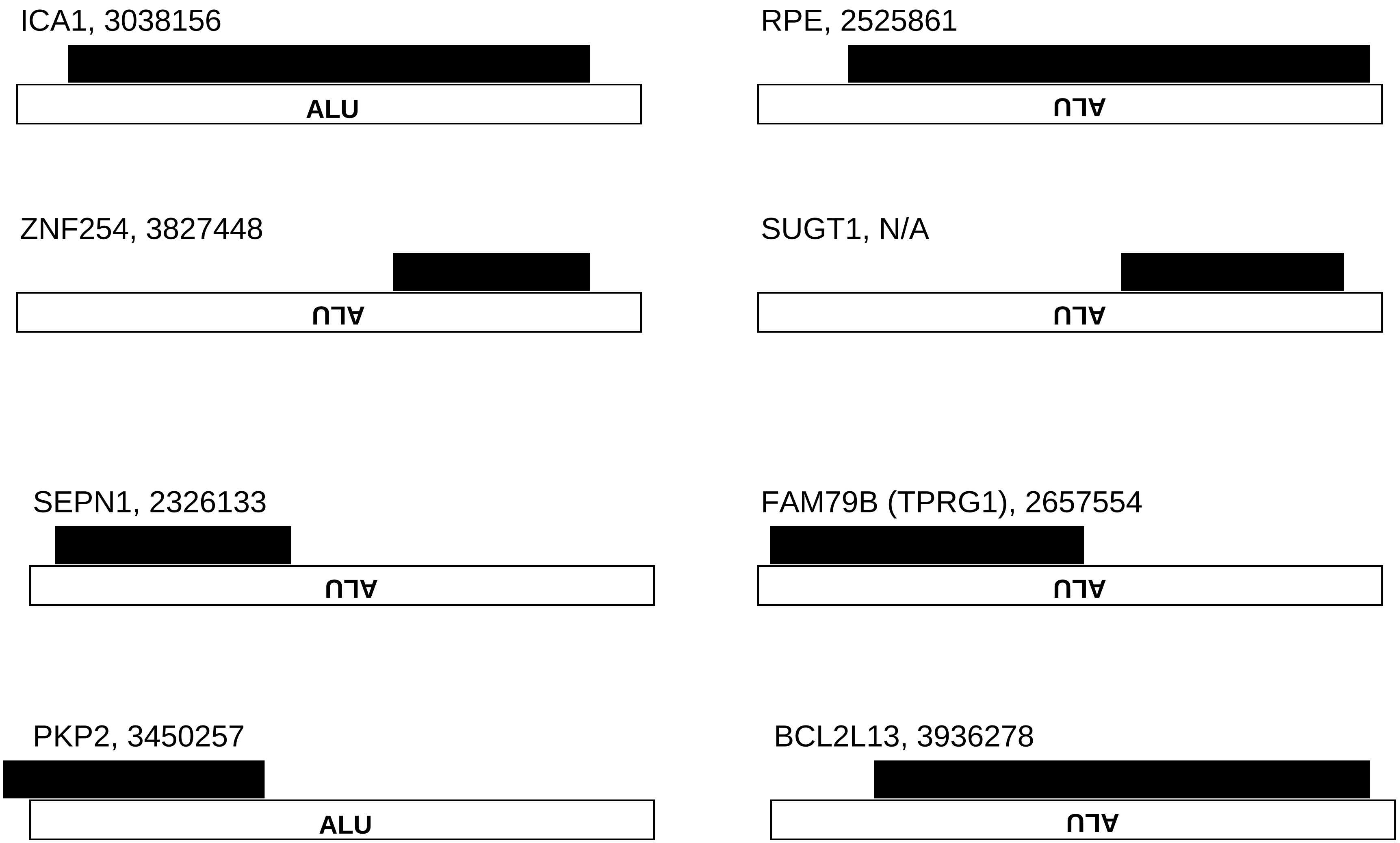
ICA1, 3038156
ALU
RPE, 2525861
ALU
ZNF254, 3827448
ALU
SUGT1, N/A
ALU
SEPN1, 2326133
ALU
FAM79B (TPRG1), 2657554
ALU
PKP2, 3450257
ALU
BCL2L13, 3936278
ALU

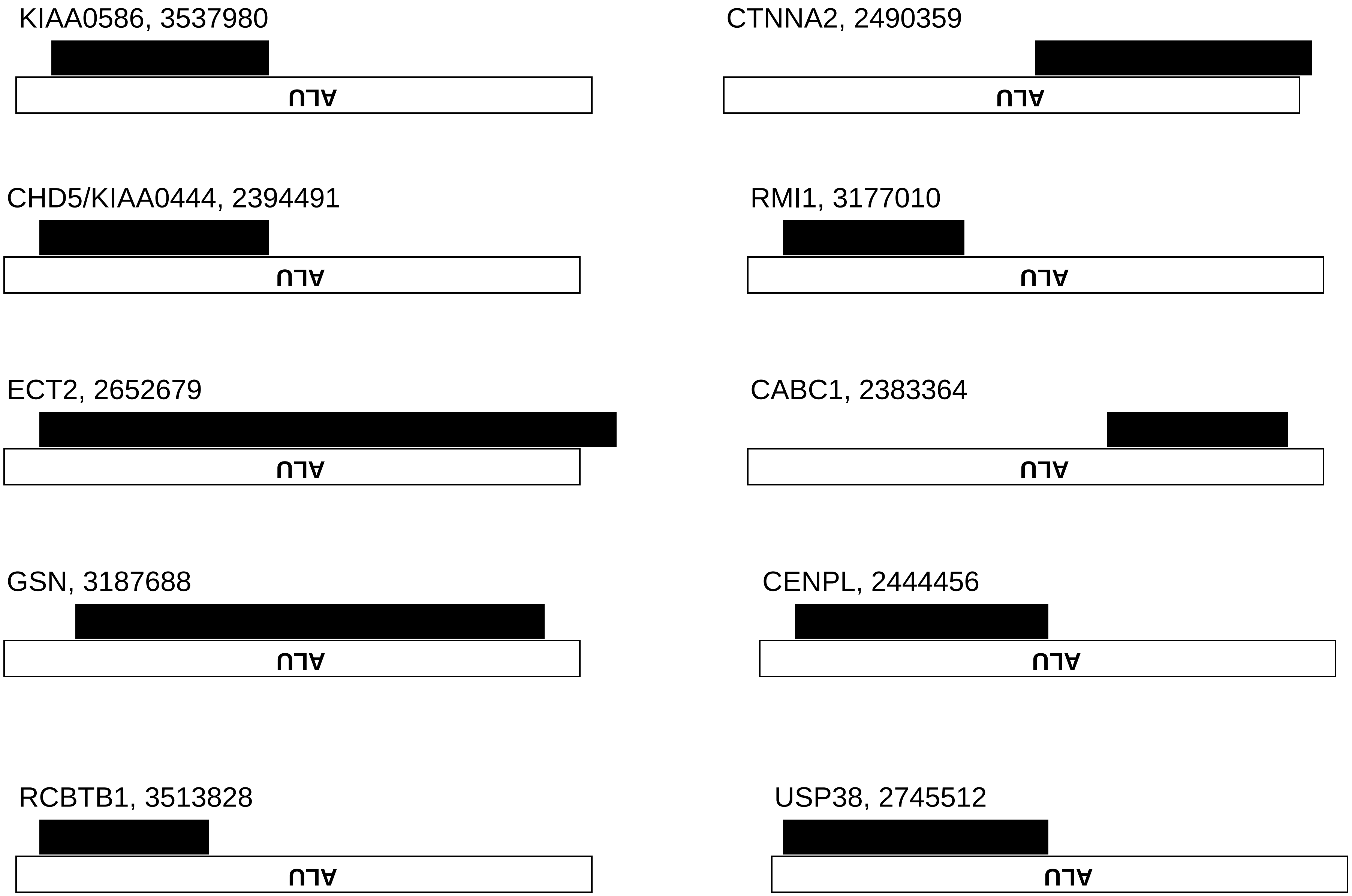
KIAA0586, 3537980
ALU
CTNNA2, 2490359
ALU
CHD5/KIAA0444, 2394491
ALU
RMI1, 3177010
ALU
ECT2, 2652679
ALU
CABC1, 2383364
ALU
GSN, 3187688
ALU
CENPL, 2444456
ALU
RCBTB1, 3513828
ALU
USP38, 2745512
ALU

FAM124B, 2601529

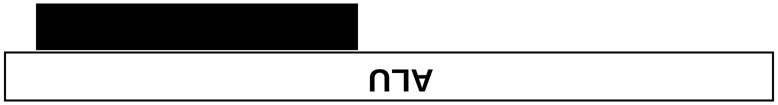

Supplement: Figure S5 — Schematic diagram of the location and orientation of the Alu-derived exons with respect to the Alu elements. Filled black box represents Alu-derived exon. Empty box represents the full-length Alu element as annotated by UCSC Genome Browser. The orientation of ‘ALU’ in the empty box represents the orientation of the exon with respect to the Alu element (sense or antisense). (0.01 MB PDF) [file pgen.1000225.s005.pdf]
