# Supplementary material for: Diverse Splicing Patterns of Exonized Alu Elements in Human Tissues
Source: PLoS Genet. 2008 Oct 17;4(10):e1000225. doi: 10.1371/journal.pgen.1000225 (PMC2562518; doi:10.1371/journal.pgen.1000225)
Supplement: Table S1 — RT-PCR analysis of Alu-derived exons whose Exon array probe intensities are uncorrelated with overall gene expression levels. (0.02 MB PDF) [file pgen.1000225.s006.pdf]

Table S1. RT-PCR analysis of Alu-derived exons whose Exon array probe intensities are uncorrelated with overall gene expression levels.

Gene	Cluster	Probeset	Target exon location	Target exon size (bp)	PCR Skipping (bp)	PCR inclusion (bp)	Alu type	Alu strand/mRNA	Splicing pattern	Impact on mRNA/protein	Gene name	GO processes/known features
**z-score <3 in 11 tissues (suggesting weak exon inclusion in all tissues).**												
FAM124B	2601499	2601529	chr2:224973342-224973466	125	197	322	AluJo	Antisense	Alternative medium form, no conclusive evidence for tissue-specificity	Alternative premature termination codon	Family with sequence similarity 124B	Unknown
KIAA0586	3537967	3537980	chr14:57977692-57977814	123	173	296	AluJb	Antisense	Alternative minor form, no tissue specificity detected	Coding	KIAA0586	Unknown
CHD5/KIAA0444	2394478	2394491	chr1:6095958-6096063	86	164	250	AluJb	Antisense	Alternative minor form, no tissue specificity detected	Coding with different reading frame	Chromodomain helicase DNA binding protein 5	Chromatin modification, DNA binidng, helicase activity
ECT2	2652675	2652679	chr3:173952634-173952956	323	138	461	AluSq	Antisense	Alternative minor form, no tissue specificity detected	5'UTR	Epithelial cell transforming sequence 2 oncogene	Positive regulation of I-kappaB kinase/NF-kappaB cascade, Rho guanyl-nucleotide exchange factor activity
USP38	2745499	2745512	chr4:144345020-144345135	116	208	324	AluJb	Antisense	Alternative minor form, no tissue specificity detected	Coding with premature termination codon	Ubiquitin specific peptidase 38	Ubiquitin-dependent protein catabolic process,cysteine-type peptidase activity
**z-score>7 in at least 3 tissues (suggesting strong or medium exon inclusion in some tissues).**												
GSN	3187686	3187688	chr9:123009899-123009996	98	None	198	AluJb	Antisense	Constitutive inclusion	5'UTR	Gelsolin	Actin filament polymerization
CABC1	2383356	2383364	chr1:225164026-225164118	93	None	266	AluJb	Antisense	Constitutive inclusion	5'UTR	Chaperone, ABC1 activity of bc1 complex homolog (S. pombe)	Cell death, transferase activity
RCBTB1	3513794	3513828	chr13:49052642-49052721	80	208	288	AluJb	Antisense	Alternative major/constitutive inclusion, no tissue specificity detected	5'UTR	Regulator of chromosome condensation (RCC1) and BTB (POZ) domain containing protein 1	DNA-dependent regulation of transcription, chromatin modification, cell cycle
CTNNA2	2490351	2490359	chr2:79278593-79278710	118	111	229/320	AluSc	Antisense	Alternative major form, no tissue specificity detected	5'UTR	Catenin (cadherin-associated protein), alpha 2	Cell adhesion
RMI1	3176999	3177010	chr9:85793064-85793139	76	185	261	AluJo	Antisense	Alternative medium form, inclusion/skipping shift in multiple tissues	5'UTR	RecQ mediated genome instability 1, homolog (S. cerevisiae)	Nucleic acid binding
CENPL	2444451	2444456	chr1:172042200-172042337	138	243	381	AluJo	Antisense	Alternative minor form, no tissue specificity detected	Coding	Centromere protein L	Required for proper kinetochore function and mitotic progression
