# Supplementary material for: Diverse Splicing Patterns of Exonized Alu Elements in Human Tissues
Source: PLoS Genet. 2008 Oct 17;4(10):e1000225. doi: 10.1371/journal.pgen.1000225 (PMC2562518; doi:10.1371/journal.pgen.1000225)
Supplement: Table S2 — Substantially included Alu-derived exons detected by RT-PCR analysis. (0.02 MB PDF) [file pgen.1000225.s007.pdf]

Table S2. Substantially included Alu-derived exons detected by RT-PCR analysis

Gene	Cluster	Probeset	Target exon location	Alu type	Alu strand/ mRNA	Splicing pattern	Impact on mRNA/protein	Gene name	GO processes/known features
FAM55C	2634058	2634065	chr3:102,984,195-102,984,314	AluJb	Antisense	Constitutive inclusion	5'UTR	Family with sequence similarity 55, member C	Unknown
NLRP1	3742783	3742834	chr17:5,377,348-5,377,428	ALuJb	Antisense	Constitutive inclusion	Coding with alternative 3' splice site	NLR family, pyrin domain containing 1	ATP binding, caspase activation, apoptosis
ZNF611	3869714	3869736	chr19:57923795-57923895	AluJb	Sense	Constitutive inclusion	5'UTR	Zinc finger protein 611	Regulation of transcription
ADAL	3591365	3591369	chr15:41412801-41412924	AluJb	Sense	Constitutive inclusion	5'UTR	Adenosine deaminase-like	Nucleotide metabolic process, deaminase activity
GSN	3187686	3187688	chr9:123009899-123009996	AluJb	Antisense	Constitutive inclusion	5'UTR	Gelsolin	Actin filament polymerization
CABC1	2383356	2383364	chr1:225164026-225164118	AluJb	Antisense	Constitutive inclusion	5'UTR	Chaperone, ABC1 activity of bc1 complex homolog (S. pombe)	Cell death, transferase activity
NOX5	3599561	3599599	chr15:67054122-67054224	AluJb	Antisense	Liver, pancreas and testes specific skipping, constitutive inclusion in other tissues	5'UTR	NADPH oxidase, EF-hand calcium binding domain 5	NADPH oxidase that generates superoxide and functions as a H+ channel in a Ca(2+)-dependent manner
RPP38	3236538	3236542	chr10:15184223-15184341	AluJb	Antisense	Constitutive inclusion, only detected in kidney and testes	5'UTR	Ribonuclease P/MRP 38kDa subunit	tRNA processing, hydrolase activity
RSPH10B	3037100	3037137	chr7:5973475-5973594	AluJb	Antisense	Constitutive inclusion, only detected in testes	5'UTR	Radial spoke head 10 homolog B (Chlamydomonas)	Unknown
CTNNA2	2490351	2490359	chr2:79278593-79278710	AluSc	Antisense	Alternative major form, no tissue specificity detected	5'UTR	Catenin (cadherin-associated protein), alpha 2	Cell adhesion
RCBTB1	3513794	3513828	chr13:49052642-49052721	AluJb	Antisense	Alternative major/constitutive inclusion, no tissue specificity detected	5'UTR	Regulator of chromosome condensation (RCC1) and BTB (POZ) domain containing protein 1	DNA-dependent regulation of transcription, chromatin modification, cell cycle
SLFN11	3753500	3753521	chr17:30718078-30718195	AluJb	Antisense	Pancreas specific minor form, alternative major form in most tissues	5'UTR.	Schlafen family member 11	ATP binding, nucleotide binding
EFCAB5	3716259	3716293	chr17:25424227-25424342	AluJo	Antisense	Alternative major form, no tissue specificity detected	Coding with alternative 3'/stop codon/3'UTR	EF-hand calcium binding domain 5	Calcium ion binding
GOLGA8A	3617458	3617512	chr15:32470616-32470919	AluSx	Antisense	Alternative major form, no tissue specificity detected	5'UTR	Golgi autoantigen, golgin subfamily a, 8A	Golgi apparatus protein
FLJ42842	3727033	3727035	chr17:46771967-46772084	AluJb	Antisense	Alternative major form, no tissue specificity detected	3'UTR	Unknown	Unknown
ADARB1	3924041	3924084	chr21:45428817-45428936	AluJb	Antisense	Alternative major form, no tissue specificity detected	Coding region, decrease catalytic activity [33]	Adenosine deaminase, RNA-specific, B1	mRNA processing, adenosine deaminase activity
FAM79B (TPRG1)	2657546	2657554	chr3:190201257-190201394	AluJo	Antisense	Alternative major form, no conclusive evidence for tissue-specificity	5'UTR	Tumor protein p63 regulated 1	Unknown
C16orf61/DC13	3701384	3701391	chr16:79571876-79571985	AluJo	Antisense	Alternative medium form, no tissue specificity detected	Exon in a non-coding transcript	Homo sapiens chromosome 16 open reading frame 61	Unknown
SHMT1	3748323	3748350	chr17:18204453-18204591	ALuJb	Antisense	Alternative medium form, no tissue specificity detected	5'UTR	Serine hydroxymethyltransferase 1 (soluble)	L-serine metabolic process, transferase activity
CLEC7A	3444009	3444018	chr12:10168953-10169039	AluJb	Antisense	Alternative medium form, no tissue specificity detected	Coding region with in frame premature termination codon OR 3' UTR	C-type lectin domain family 7, member A	T-cell activation, inflammatory response, MHC protein binding
ICA1	3038065	3038156	chr7:8233793-8234006	AluJo	Sense	Testes specific inclusion	Premature stop or alternative start	Islet cell autoantigen 1	Neurotransmitter transport, autoantigen in insulin-dependent diabetes mellitus and primary Sjogren's syndrome
ZNF254	3827427	3827448	Chr19: 24023579-24023656	AluJb	Antisense	Cerebellum specific major form, pancreas specific skipping, alternative medium form in other tissues	5'UTR	Zinc finger protein 254	DNA binding, negative regulation of transcription from RNA polymerase II promoter
SEPN1	2326126	2326133	chr1:26001094-26001195	AluJb	Antisense	Muscle specific major form, alternative minor form in most tissues	Coding , but no protein detection in previous report	Selenoprotein N, 1	Calcium ion binding, mutations in this gene cause the classical phenotype of multiminicore disease and congenital muscular dystrophy with spinal rigidity and restrictive respiratory syndrome.
SUGT1	N/A	N/A	chr13:52133611-52133706	AluSx	Antisense	Alternative medium form, no conclusive evidence for tissue-specificity	Coding	Suppressor of G2 allele of SKP1	Mitosis
FAM124B	2601499	2601529	chr2:224973342-224973466	AluJo	Antisense	Alternative medium form, no conclusive evidence for tissue-specificity	Alternative premature termination codon	Family with sequence similarity 124B	Unknown
RMI1	3176999	3177010	chr9:85793064-85793139	AluJo	Antisense	Alternative medium form, inclusion/skipping shift in multiple tissues	5'UTR	RecQ mediated genome instability 1, homolog (S. cerevisiae)	Nucleic acid binding
