# Supplementary material for: Diverse Splicing Patterns of Exonized Alu Elements in Human Tissues
Source: PLoS Genet. 2008 Oct 17;4(10):e1000225. doi: 10.1371/journal.pgen.1000225 (PMC2562518; doi:10.1371/journal.pgen.1000225)
Supplement: Table S3 — RT-PCR primers and PCR product sizes of all tested Alu-derived exons. (0.06 MB PDF) [file pgen.1000225.s008.pdf]

Table S3. RT-PCR primers and PCR product sizes of all tested Alu-derived exons.

Gene name	Alu exon size (bp)	Predicted skipping form (bp)	Predicted inclusion form (bp)	Forward primer	Reverse primer
ADAL	124	232/332/333	356/456/457	GGGAAACCTTGCCTAAGTCC	GCTGTTGCTCTTCTGCCTCT
ADARB1	120	142	262	GGCTGAAGGAGAATGTCCAG	GTCCGTAGCTGTCCTCTTGC
B3GALNT1	119	158/253/251/346	277/372/370/465	GCATGGAATACAGAAAACAACAA	AGGCTCCATTTGAGGGATCT
BCL2L13	98	152	250	TGAAGAAGAGCTAAAATCTCTGGAC	CCTGGGACACTTTTTCTCCA
CABC1	93	173	266	GGCAAAACAAATGGCAGAGT	AACCAAGATTGCACCACACA
CAMKK2	123	133/197	256/320	CCCTCCAGGTCTTCGTTTCT	CAGCTTCATCCAGCACACTG
CCDC53	103	147	250	TGCCAGCTATTCAACAGAAAA	TGAGAGTTGTTTCAATTTGTTGG
CENPL	118/138	243	361/381	AGGACTTGCTGTGGAAGTGG	GCAAGGAATAAGGGCAGACA
CHD5/KIAA0444	106	164	270	CGCCTTGGATAGAGTGGAGA	CCAGCTTGTCCAGGATCTTC
CLEC7A	87	103/222	190/309	AAAGACAATGCTGGCAACTG	TTGGAGATGGGTTTTCTTGG
CTNNA2	118	111	229	CTCAAATCTTCACCGGCTGT	TCATGCATCTCATCTTTGCAG
DC13	110	152	262	TGATGTTGATCGGGAGTTGA	CCAAGGCATCGAGTGAAAAT
ECT2	323	138	461	ATGGCGGTATTTGTGAGAGG	GCCAAGCTAGTCCTCCCAGT
EFCAB5	116	197	313	CAGGCTTCCTGGATCTGAAG	GGAGTATGGGATGCATAGGG
FAM124B	125	197	322	TCAGGACTACGATGGCAACA	CAGGAAGCTCCAGAGAATGC
FAM55C	120	130	250	TGCTGGAAGGAAAGTTAGACG	CAGAGGAGAGACTGCAGTTGA
FAM79B	138	153	291	TAAAGGCCTGGGGATCTTCT	TCTGCTCCATGCAGTCATTC
FLJ42842	118	198	316	TGACCTGTGGTGCTTGTGAT	CCTGCCAGGTCACAGAATTT
GOLGA8A	142/228/229	178	320/406/407	ACTCGACAGAACCGGTCATC	CGCTGATCAACACATCCAGT
GSN	98	100	198	ACACGCCCCTCTGAAGAAC	CCTGGTTTCAGTGAGGAAGG
ICA1	214	156	370	TCAATTCAGAGAACCTGTCTGG	TGTCGCTTGCATCATCTTTC
KIAA0586	123	173	296	CAATGGGACAGAAAGATGCTC	AGCCACGGTTGTAGCTGAAT
MIPOL1	139	230/357/343/470	369/496/482/609	GATCGTCTGTGGGTGAGTCT	CTGTAGTAAGCTTCTCCTTTTCCA
NLRP1	93	115	208	CCTGGATACGGGAGAGATGA	AGAAGGCACGCACAAGAGTT
NOX5	103	128	231	CCTTCTAGTTGCGCTTTTGC	CCATCTTCTCCTGCAATGGT
PKP2	117	196	313	GTGCTGAAGCAAACCAGAGA	TGTCAAAATCGAGCAAACCA
RCBTB1	80	208	288	CCATTGGAGCTTCGGAGA	AGTGCTTCACTGGCTGAGGT
RMI1	76	185	261	CCAGACTTATGCCCGTGTTT	AGGAACAGCAGCACAGGATT
RPE	54	233	287	GCCAGAACAGTGGGTAAAGC	TGAATTTCTGCCCTCCAAAC
RPP38	119	145	264	TTCTGTTCTCCACGGTCTCC	CCAGAGGTCTCGTCTTACGG
RSPH10B	120	214	334	CGAGGTCTCTGGGCTCCT	TGTTGCCATCTTGTTTGGAA
SEPN1	102	127	229	ATCAGCCCTGAGGAGTTCAA	GAATCGGGCTTCTATGGTGA
SHMT1	139	148	287	GTCAGCGGGTCTGGGACT	TTGTGCCAGCATCTTGTCAT
SLFN11	118	156/254	274/372	GCTGGAGCTTGAGAGGTCAG	TGATTTGCCTCCATGTTGAA
SUGT1	96	98	194	TGCTGCTGCCCTAGAAACTT	CATTCTGAGCTTCTTGACACCTT
USP38	116	208	324	TGGTCTGCCTTCAAGTACAGC	AGACAAGCAAGACGCCAAAG
ZNF254	78	190	268	AGAGCCCTGGACTGTGAAGA	TCAATTCTGGGTCCAACGAT
ZNF611	101	103/124/155/176	204/225/256/277	ATTGCTGTATTGCCTGGTGA	TTCATCAATTGCCATCCAGA
