# Supplementary material for: Diverse Splicing Patterns of Exonized Alu Elements in Human Tissues
Source: PLoS Genet. 2008 Oct 17;4(10):e1000225. doi: 10.1371/journal.pgen.1000225 (PMC2562518; doi:10.1371/journal.pgen.1000225)
Supplement: Text S1 — Supplemental methods. (Exon-centered genomic scan for positive selection in ADARB1 and p75TNFR). (0.10 MB PDF) [file pgen.1000225.s009.pdf]

Supplemental Methods.

Exon-centered genomic scan for positive selection in ADARB1 and p75TNFR.

We searched for evidence of positive selection on Alu-derived exons of ADARB1 and p75TNFR by identifying the signatures of selective sweep [1] in the regions flanking the exons. A recent selective sweep produces distinct signatures in the genomic variation, such as, a reduction in the amount of variation or heterozygosity, a skew in the distribution of allele frequencies towards more alleles of low frequencies, and a temporary increase in the strength of linkage disequilibrium [1-5]. We also searched for the increased levels of genetic differentiation among populations within the exons, a signature of local adaptation—positive selection may increase levels of genetic differentiation among populations exhibiting higher F_{ST} values [6-11].

Specifically, we used the exon-centered sliding window approach to scan human genome for selection pressure. For each exon, we downloaded HapMap (I+II) SNPs [12,13] located within the 2 Mbp regions around it, and then used the sliding window of 20 SNPs (with the step of one SNP) to scan the region. For each window, we excluded coding SNPs and computed values of heterozygosity, Tajima's *D* [14], Fay and Wu's *H* [15] from all noncoding SNPs. The heterozygosity for a window was calculated as the arithmetic average of heterozygosities of the SNPs in that window. The estimator for heterozygosity of the *i*-the SNP was $2p_i(1-p_i)[n/(n-1)]$, where *n* is the sample size, and p_i the allele frequency of the SNP. The *D* and *H* statistics were computed by using functions in PGEToolbox [16]. To compute *H*, we determined the ancestral state for each SNP by using the alignments between human and chimpanzee genome sequences: if an allele of a human SNP is identical to the base of the aligned chimpanzee sequence, then the allele was regarded as the ancestral allele. Some SNPs whose ancestral states could not be determined (for example, either allele of a human SNP differs from the base of chimpanzee sequence, or the chimpanzee base is undetermined) were excluded. Values of *H* were normalized with the method of [17] and validated with an independent implementation by J. Fay (personal communication to J. Cai). The sliding window analysis was repeated with SNPs from both CEU and YRI populations. We computed F_{ST} between CEU and YRI populations for all available SNPs within the region containing the exon. The function `snp_fst.m` in PGEToolbox [16] was used to calculate the unbiased estimates of F_{ST} [18,19], as described in [7].

Reduction in heterozygosity, *D* or *H* was taken as putative signature of a selective sweep and increment of F_{ST} values was considered as indication of local adaptation. To assess the significance of reduction or increment of these statistics, we obtained the empirical distributions of these statistics from the genome-wide resamplings. We randomly selected 1,000 constitutively spliced exons in the human genome. For each of these exons, we conducted the same exon-centered sliding window analysis and calculated all the statistics for both populations. Thus, for all positions around an exon, we have the distributions of these statistics. From these distributions, we calculated the *P*-value for each observed statistic in the spatial profile of the exon. Figure S1 shows the negative natural log of *P*-values for the Alu-derived exon in ADARB1 (A) and p75TNFR (B).

1. Maynard Smith J, Haigh J (1974) The hitch-hiking effect of a favourable gene. Genet Res 23: 23-35.
2. Kaplan NL, Hudson RR, Langley CH (1989) The "hitchhiking effect" revisited. Genetics 123: 887-899.
3. Stephan W, Song YS, Langley CH (2006) The hitchhiking effect on linkage disequilibrium between linked neutral loci. Genetics 172: 2647-2663.
4. Barton NH (1998) The effect of hitch-hiking on neutral genealogies. Genetics Research 72: 123-133.
5. Braverman JM, Hudson RR, Kaplan NL, Langley CH, Stephan W (1995) The hitchhiking effect on the site frequency spectrum of DNA polymorphisms. Genetics 140: 783-796.
6. Lewontin RC, Krakauer J (1973) Distribution of gene frequency as a test of the theory of the selective neutrality of polymorphisms. Genetics 74: 175-195.
7. Akey JM, Zhang G, Zhang K, Jin L, Shriver MD (2002) Interrogating a high-density SNP map for signatures of natural selection. Genome Res 12: 1805-1814.
8. Beaumont MA (2005) Adaptation and speciation: what can F(st) tell us? Trends Ecol Evol 20: 435-440.
9. Thornton KR, Jensen JD (2007) Controlling the false-positive rate in multilocus genome scans for selection. Genetics 175: 737-750.
10. Beaumont MA, Balding DJ (2004) Identifying adaptive genetic divergence among populations from genome scans. Mol Ecol 13: 969-980.
11. Pollinger JP, Bustamante CD, Fledel-Alon A, Schmutz S, Gray MM, et al. (2005) Selective sweep mapping of genes with large phenotypic effects. Genome Res 15: 1809-1819.
12. International-HapMap-Consortium (2003) The International HapMap Project. Nature 426: 789-796.
13. International-HapMap-Consortium (2007) A second generation human haplotype map of over 3.1 million SNPs. Nature 449: 851-861.
14. Tajima F (1989) Statistical method for testing the neutral mutation hypothesis by DNA polymorphism. Genetics 123: 585-595.
15. Fay JC, Wu CI (2000) Hitchhiking under positive Darwinian selection. Genetics 155: 1405-1413.
16. Cai JJ (2008) PGEToolbox: A Matlab toolbox for population genetics and evolution. J Hered 99: 438-440.
17. Zeng K, Fu YX, Shi S, Wu CI (2006) Statistical tests for detecting positive selection by utilizing high-frequency variants. Genetics 174: 1431-1439.
18. Weir BS, Cockerham CC (1984) Estimating F-statistics for the analysis of population structure. Evolution 38: 1358-1370.
19. Weir BS (1996) Genetic Data Analysis II. Sunderland, Massachusetts: Sinauer.
